# Mathematical Models Generated for the Prediction of Corrosion Inhibition Using Different Theoretical Chemistry Simulations

**DOI:** 10.3390/ma13245656

**Published:** 2020-12-11

**Authors:** José A. Rodríguez, Julián Cruz-Borbolla, Pablo A. Arizpe-Carreón, Evelin Gutiérrez

**Affiliations:** 1 Area Académica de Química, Universidad Autónoma del Estado de Hidalgo, Unidad Universitaria, km 4.5 Carretera Pachuca-Tulancingo, Pachuca-Hidalgo C.P. 42184, Mexico; josear@uaeh.edu.mx (J.A.R.); jcruz@uaeh.edu.mx (J.C.-B.); 2 Ingeniería Aeronáutica, Universidad Politécnica Metropolitana de Hidalgo, Boulevard Acceso a Tolcayuca 1009, Ex Hacienda San Javier, Tolcayuca C.P. 43860, Mexico; parizpe@upmh.edu.mx

**Keywords:** corrosion inhibition, organic inhibitors, theoretical studies, molecular descriptors, mathematical models

## Abstract

The use of corrosion inhibitors is an important method to retard the process of metallic attack by corrosion. The construction of mathematical models from theoretical-computational and experimental data obtained for different molecules is one of the most attractive alternatives in the analysis of corrosion prevention, whose objective is to define those molecular characteristics that are common in high-performance corrosion inhibitors. This review includes data of corrosion inhibitors evaluated in different media, the most commonly studied molecular descriptors, and some examples of mathematical models generated by different researchers.

## 1. Introduction

Corrosion is defined as the destructive attack of a material by reaction with its environment. This process is a worldwide, significant problem because of the economic damage and safety loss it may lead to. The losses originating from corrosion can be categorized as direct and indirect, and the latter includes economic losses caused by plant shutdowns, efficiency reduction, costly maintenance, and contamination of products whose overall impact will ultimately require over-design. The direct losses include the cost of replacing corroded structures, fixing damaged machinery, and substituting some of its components [1].

Corrosion control can be achieved by recognizing and understanding the mechanisms associated with this process. It is possible to prevent corrosion by using corrosion-resistant materials and protective systems.

The selection of materials; which can be metallic, non-metallic or alloys, is a key factor in the prevention of corrosion processes, as well as the consideration of the temperature, the type of environment and the general conditions to which the material is subjected. In such selection, both the mechanical and physical analyses of the materials’ properties play a very important role. However, such selection is limited by the availability and cost of materials. This limitation forces the industry to resort to the corrosion protection offered by metallic or non-metallic coatings and anodic or cathodic processes for metal protection, as well as to carefully select the appropriate geometrical configurations to prevent corrosive conditions and to avoid the use of bimetallic couples, which can be responsible for favoring the corrosion process. Corrosion protection can also be achieved by promoting the formation of Ni and Cr protective barriers or by protecting a substrate (steel) with sacrificing materials such as Zn, Al or Cd [2].

As was previously mentioned, the environments to which metals are exposed make them prone to corrosion. A less aggressive environment can be obtained by removing constituents that facilitate corrosion, modifying the temperature, dehumidifying the air, removing dissolved O_2_ or solid particles, controlling the pH or adding corrosion inhibitors [2]. The usage of inhibitors is one of the most practical methods for protection against corrosion in acidic, alkaline, saline, and other aggressive environments [3,4].

The following sections include:

- The definition of a corrosion inhibitor and the structural characteristics associated with an inhibitor molecule; a brief mention is made of the techniques that allow evaluating the performance of corrosion inhibitors in the protection of metal surfaces exposed to aggressive environments.
- The factors that increase the corrosion process and those that can be modified to prevent this phenomenon are briefly mentioned.
- The most important data such as molecules and inhibition efficiencies obtained by different techniques and experimental conditions have been summarized in this manuscript, taking into account the functional group of greatest interest to the study authors.
- Information related to the descriptors used in the theoretical analysis of corrosion inhibitors has been collected.
- Finally, a table has been generated with examples of some mathematical models obtained to evaluate corrosion inhibitor molecules, in which it is possible to observe the most common descriptors to predict the corrosion phenomenon.

## 2. Corrosion Inhibitors

Corrosion inhibitors are chemical substances that are added to aggressive environments in small concentrations to decrease the corrosion rate. These substances can react with either a metal surface or its surroundings providing protection to the surface, although it has been observed that corrosion inhibitors generally work by forming an adsorbed film. Corrosion inhibitors are an appropriate option for metal protection when these are exposed to aggressive media, such as, acidic solutions (widely employed for industrial cleaning), oil well acidification, and petrochemical processes [5]. Corrosion inhibitors can be introduced into the aggressive environment in a single application or continuously by gradual and controlled additions. The single addition is possible in static systems at low temperature and where friction is negligible. Gradual addition is necessary for systems where variation in flow and temperature degrade the integrity of the protective film formed on the metal surface, either by physisorption or chemisorption. The stability of the corrosion inhibitor films can be compromised by the concentration levels of the inhibitor in the medium [6]. Comparative studies performed to determine inhibition effects are generally performed without shaking. Therefore, the existence of a significant effect of immersion time on the quality of the formed film of an inhibitor is reasonable.

An important number of compounds have been used for the corrosion protection of metals exposed to aggressive media. Their effectiveness is associated to their chemical composition, molecular structure, alkyl chain length (or molecular volume), planarity, presence of lone pairs of electrons in heteroatoms (e.g., S, N, O, P), affinity for the metallic surface, dipole moment, presence of π-electrons (unsaturation or aromatic ring) and energy of frontier molecular orbitals. The efficiency (*E*%) of corrosion inhibitors is measured by applying the Equation (1) to experimental data obtained by techniques such as weight loss (WL), potentiodynamic polarization (PP), and electrochemical impedance spectroscopy (EIS):

(1) $$E\%=\frac{\left(CR_{u}-CR_{i}\right)}{CR_{u}}\times 100$$

where *CR_u_* is the corrosion rate of the uninhibited system and *CR_i_* is the rate of the inhibited system.

The mechanisms through which corrosion inhibitors can be bounded to the metal or the metal oxide surface may be physisorption, chemisorption, complexation or precipitation. The film formed by corrosion inhibitors prevents the access of oxygen to the cathode and the diffusion of hydrogen away from it, or simply inhibits metal dissolution (anodic inhibitors). Inhibition efficiency can be altered by modifying the system parameters such as pH, temperature, metal composition, type of inhibitor and molecular structure of the corrosion inhibitor [7]. An important factor associated with the corrosion process is surface roughness. The increase in the surface roughness of alloys (magnesium, titanium-based), stainless steels, copper, and aluminum increases the pitting susceptibility and the corrosion rate [8].

Corrosion inhibitors can act by stimulating anodic or cathodic polarization, reducing the transport of ions towards the metallic surface and increasing the electrical resistance of a metallic material. These compounds can be classified according to their functionality (anodic, cathodic, organic and precipitation-inducing inhibitors). Anodic inhibitors are molecules that cause a large anodic shift of the corrosion potential and by this means they are able to generate the passivation of the metal surface. On the other hand, cathodic inhibitors either slow the cathodic reaction or selectively precipitate on cathodic areas increasing the surface impedance and reducing the diffusion of species. Some cathodic inhibitors may precipitate as oxides building a protective layer on the metallic surface. Organic inhibitors can be classified as another type of inhibitor. These substances can be associated with anodic and cathodic effects; they generally protect the metallic surface by creating a film whose function consists in isolating the metal from corrosion. Finally, precipitation-inducing inhibitors are compounds responsible for the formation of precipitates on the metallic surface which protect it from the corrosion process [2,9]. In the study of amino acids carried out by Aouniti et al. [10], it was demonstrated by the analysis of cathodic and anodic potentiodynamic polarisation curves, that these compounds act as cathodic inhibiting only the cathodic process. In the analysis, before recording the cathodic polarisation curves, the electrode was polarised at −800 mV/SCE (saturated calomel electrode) for 10 min, while for the anodic curves, the potential electrode was swept from its corrosion potential (maintained 30 min) to positive values. No changes were observed in the anodic curves.

The phosphonate derivatives evaluated by Benabdellah et al. [11] are also cathodic inhibitors that cause a decrease in current density and do not modify the characteristics in the anodic domain. In the study, the slopes of cathodic Tafel lines and the corrosion potential remain almost constant which reveals that the mechanism of reduction of hydrogen ion is not affected by the presence of inhibitor. An important number of corrosion inhibitors act as mixed inhibitors blocking the cathodic reaction sites (hydrogen evolution) and anodic reaction sites (metal dissolution) on the metallic surface [12].

There are also mixed inhibitors with the most significant anodic effect, which can be observed in the change of the slopes of anodic Tafel lines and the displacement of corrosion potential towards positive values compared with not protected surfaces. Among these, hydantoin [13], pyrimidine [14], and Schiff base compounds [15] are also reported. Film-forming compounds are precipitation-inducing inhibitors. Precipitation inhibitors act on both the anodic and cathodic sites indirectly by blocking the metal surface, examples include silicates and phosphate [16]. As has been previously mentioned, inhibitors often act by adsorbing themselves on a metallic surface preventing the corrosive attack through the formed film. The adsorption of corrosion inhibitors depends on the charge of the corrosion inhibitor and the charge of the metallic surface, as well as the temperature and pressure involved in the process. Many researchers have mentioned that compounds containing nitrogen, oxygen and sulphur atoms give rise to a high inhibition efficiency in acidic media [17,18,19]. Most organic substances containing at least one functional group that can serve as a potential reaction centre for adsorption on a metallic surface can be probed as a corrosion inhibitor [20]. 

Corrosion protection as a result of a film formation process is mainly associated with sorption processes. The organic compounds adsorbed on a metal surface replace the pre-adsorbed water molecules and prevent the dissolution of the metallic surface, reducing the interaction between the metal surface and corrosive electrolytes [21].

Ehteshamzadeh et al. [15] described the interaction processes of organic corrosion inhibitors and a metallic surface through four mechanisms: (a) electrostatic interaction between a negatively charged surface, which is a result of the (Cl^−^) anions adsorbed on iron and the positive charge of the inhibitor in HCl media, (b) interaction of the unshared electron pairs of the corrosion inhibitor with the metallic surface, (c) interaction of π-electrons with the metal and (d) the combination of types a-c. The adsorption process can be named physisorption only when electrostatic forces between ionic charges or dipoles on the adsorbed species and an electric charge at the metal/solution interface are involved. This adsorption mechanism has low values of adsorption heat and it is stable only at low temperatures. The adsorption process can also occur through chemisorption, which involves charge sharing or charge transfer from the corrosion inhibitor to the metal surface forming a coordinate-type bond. Chemisorption is associated with a higher adsorption energy than physical adsorption, therefore the established bond will be stable at higher temperatures [5,22,23].

Among the compounds proposed as corrosion inhibitors we find: N-containing heterocyclic organic molecules such as imidazole, benzimidazole, bis-benzimidazoles, amine, Schiff base compounds and alkaloids (e.g., papaverine, strychnine, quinine, and nicotine); S-containing compounds that comprise sulphonamides-which possess a large number of functional groups that are potential adsorption centres (-NH_2_ group, -SO_2_-NH- group, O and/or N heteroatoms and aromatic rings)-, sulfonates, thiols, and thioaldehydes; O-containing compounds that can include benzoates, aldehydes, indanone derivatives; and finally, P-containing compounds as phosphonate derivatives and heteroatom-containing compounds such as azoles. The studies reveal that the inhibition of corrosion processes is mainly attributed to the formation of donor-acceptor complexes [11,12,18,19,21,24].

According to observations performed in presence of compounds with heteroatoms in their structure, the corrosion inhibition efficiency follows this tendency: O < N < S < P. This behaviour is also associated with the electronegative character of the involved atoms [11,25]. There is a growing interest in defining the best molecular characteristics of a corrosion inhibitor in addition to the effects associated with the presence of heteroatoms, while also taking factors such as cost, toxicity, availability and environmental friendliness into account [2].

The following sections contain a review of some of the molecules studied as corrosion inhibitors in different environments. In the cases where the number of compounds evaluated by the authors was larger than four, only the best two and the worst two corrosion inhibitors are included, there is also mentioned the total number of corrosion inhibitors (N_CI_) employed in every analysis. For almost all of the studies reviewed in this paper, the corrosion inhibition efficiencies tabulated correspond to the values obtained at the highest inhibitor concentration. It is worth noting that the most efficient corrosion inhibitors tended to be complex molecules with more than one heteroatom in their structure; however, for the purpose of this review, corrosion inhibitors have been classified according to the heteroatom considered of higher interest by the referenced researchers.

The following sections summarize the analysis conditions employed by different researchers and the corrosion inhibition efficiencies obtained by the different techniques; weight loss (Ef_WL_), potentiodynamic polarization (Ef_PP_), and electrochemical impedance spectroscopy (Ef_EIS_), at those conditions. Cases where the theoretical analysis (TA) was applied (A) or not (NA) are mentioned. There is an important number of experimental conditions (Exp.C.) that affect the corrosion inhibition efficiency, among these factors we find: the metallic surface (Met) and the exposure time (t) of this in inhibited and uninhibited solutions, temperature (T), aggressive media and its concentration (Media), as well as the corrosion inhibitor concentration (Co), nature of the anion in the corrosive medium, type of metal, and pH [26,27]. The immersion time (a) can affect the natural oxide film of some metallic surfaces, (b) can be responsible for increasing the thickness of the layer formed by corrosion products, which can reduce the corrosion rate, (c) may favour or not the increase of corrosion inhibitor adsorption leading to the formation of uniform and stable films of corrosion inhibitors.

## 3. N-Containing Corrosion Inhibitors

An important number of molecules evaluated as corrosion inhibitors in acidic media contain N-atoms and the structural properties that are characteristic of high-performance corrosion inhibitors. Table 1 shows some examples of the N-containing corrosion inhibitors evaluated by different researchers. At least four examples of the total molecules evaluated have been included. In the second column, those molecules that were represented graphically have been marked with an asterisk.

Ashassi-Sorkhabi et al. [3], studied the effect of three Schiff base compounds as corrosion inhibitors in mild steel exposed to acidic media (HCl 1.0 M). The structure of the Schiff bases, with the presence of a benzene ring and -C=N group, is associated with the formation of an important π bond, which can be considered a good characteristic in a corrosion inhibitor. The π electrons of Schiff base compounds interact with unoccupied orbitals of iron, and also the π* orbital can accept electrons coming from the d orbitals of iron, resulting in the formation of feedback bonds, giving place to adsorption centres. Researchers observed that the inhibition efficiency depended on the type of functional groups attached to the benzene ring and demonstrated that the presence of electron-donating groups (methyl group and chloride) increased the effect of the corrosion inhibitor benzylidene-pyridine-2-yl-amine, and this happened as a result of the increase in the electron density on the nitrogen atom of the C=N group. Gomma et al. [32] performed the analysis of Schiff bases as corrosion inhibitors for aluminium (commercial-grade) in hydrochloric acid solution. Although aluminium possesses a passive oxide film that protects it in various environments, this film is amphoteric and can be dissolved when exposed to acidic media (pH lower than 5) or alkaline media (pH higher than 9) [33]. The corrosion inhibitors evaluated by Gomma et al. [32] caused a displacement of the corrosion potential in the negative direction, which means that the inhibitors employed are cathodic. It was observed that the inhibition efficiency increased as temperature rose, which makes these inhibitors an interesting option to delay the corrosion at elevated temperature conditions. Such behaviour is associated to a diffusion process, in which at a higher temperature the amount of inhibitor that reaches the metallic surface is greater than under lower temperature conditions. Higher temperatures are associated with higher activation energy available for adsorption processes, higher diffusion rate of inhibitor molecules and changes in the inhibitor molecule, causing increases in the electron density in the vicinity of the adsorption centres which, in turn, increases the efficiency [37].

For many other compounds it has been found that increases in temperature can be responsible for the desorption of the inhibitor molecules from the metallic surface, decreasing the inhibition efficiency [33].

Like Schiff bases, molecules such as triazole derivatives have an important number of N atoms in their structures. The substitution of these represents an important change in corrosion inhibition. It is considered that triazole compounds reduce corrosion rates and hydrogen embrittlement of metals such as steel and copper in acidic media. Guo et al. [25] carried out theoretical studies to correlate the corrosion inhibition effect of three triazole derivatives with quantum chemical parameters obtained by density functional theory (DFT). The triazole derivatives evaluated reached corrosion inhibition efficiencies between 90% and 97% in HCl 1.0 M media. Bentiss et al. [38] described the adsorption of a triazole derivative, which takes place either as a result of the donor-acceptor interactions between the π-electrons of the heterocycle compound and the vacant d-orbitals of the iron atoms, or by the interaction with the previously adsorbed chloride or sulphate ions when the aggressive medium consists of HCl or H_2_SO_4_. A series of constructed models from theoretical information demonstrated similarity when molecules are evaluated in aqueous phase and gas phase. However, through quantum chemical calculus it was observed that the solvation of inhibitors causes significant changes in the charge distribution. One conclusion from this study is that the results may be used to propose possible routes to modify corrosion inhibitors to achieve high performance [25].

The corrosion inhibition of aluminium exposed to HCl solution was studied experimentally and theoretically using three oxime compounds, which are good cathodic inhibitors characterized by the functional group C=N-OH which includes simultaneously N, O and a double bond. These compounds are considered non-toxic and biodegradable and possess the advantage of having a good solubility. The corrosion inhibition rates obtained at an inhibitor concentration of 2.0 mM were 10.25, 6.68 and 3.13 g⋅m^−2^⋅h^−1^ vs. 51.26 g⋅m^−2^⋅h^−1^, being this latter the corrosion rate in the absence of inhibitor. Generally, corrosion inhibitors obey the Langmuir adsorption isotherm:

(2) $$\frac{c}{\theta }=\frac{1}{K}+c$$

where *c* is the inhibitor concentration, *K* is the adsorption equilibrium constant, and *θ* is the surface coverage. In the analysis of oxime compounds it has been observed that a large value of *K* is related to the easy, strong adsorption of the inhibitor on the metallic surface, thus high values of *K* are associated to a better inhibition performance. The effect of oxime compounds in the corrosion inhibition of aluminium can be attributed to the formation of coordination bonds between the unshared electron pairs of the O or N atoms (associated to the C=N-OH functional group) and the empty p-orbital of the aluminium atom. The use of oxime compounds for aluminium protection demonstrated that the inhibition efficiency of these compounds decreases with the temperature [33].

Bentiss et al. [28] synthesized two oxadiazoles derivatives to be evaluated as corrosion inhibitors; these compounds are considered an attractive alternative to corrosion protection, mainly due to the abundance of π-electrons and unshaped electron pairs on the heteroatoms present in their structure, which can interact with d orbitals of iron and to provide a protective film. Oxadiazoles inhibit the corrosion processes in HCl and H_2_SO_4_; however, better performance has been observed in HCl presence. The results obtained in HCl are displayed in Table 1. The effect in HCl media can be the result of the presence of oxadiazole as either a neutral molecule or its cationic form making the adsorption possible by displacing the water molecules and sharing the electrons of its nitrogen atoms with the metallic surface, or by attaining the adsorption through electrostatic interactions between the positive nitrogen atom (cationic form) and the negatively charged metal surface. The worst performance observed in H_2_SO_4_ medium can be associated with the presence of SO_4_^−^ ions strongly adsorbed on mild steel which make the adsorption of organic molecules difficult. According to different authors, molecules with heteroatoms, such as nitrogen, are effective as corrosion inhibitors; their effect is increased as a result of the presence of aromatic rings and different substituents. It has been reported that corrosion inhibitors are present as protonated species in acidic media and such protonated species can be attracted by acid anions (SO_4_^−^ or Cl^−^) of the negatively-charged metal surface [24].

Small molecules as imidazole have been reported to have low corrosion inhibition efficiencies (17.5%); Bereket et al. [30] carried out quantum chemical studies using some imidazole derivatives. In their study, the conditions or the technique used were not specified; however, it is possible to observe that molecules such as 1,2-bisbenzylbenzimidazole have high corrosion inhibition efficiencies (96%) on iron exposed to HCl 1.0 M, this can be attributed to the two anchoring sites suitable for surface bonding: the nitrogen atom and the aromatic rings in the benzimidazole molecule [5]. The molecules reported by Bereket and co-workers show that heterocyclic compounds with polar groups and/or π electron cloud reduce the corrosive attack of metals in acidic media. In this study these researchers divided the studied molecules into series to undertake systematic studies on structure and inhibition efficiencies; they talk about the possibility of parallel adsorption mode which can be associated to the interaction between the positively charged iron in acidic solution and negatively charged centres of molecules. The parallel mode adsorption explained should be key to the correlation between calculated total charges of each molecule versus inhibition efficiencies.

Dutta et al. [12] performed the analysis of four bis-benzimidazole derivatives for mild steel corrosion protection. The compounds evaluated demonstrated good inhibition performance for a long period of exposure. It was observed that the inhibition efficiency is higher when two benzimidazole moieties are separated by groups having a lone pair of electrons or π electron cloud. In the 2,6-bis-(2-benzimidazolyl) pyridine compound, the lone pair of electrons on N and the π electron cloud of pyridine are available sites for interaction with the metallic surface improving its performance as a corrosion inhibitor. The presence of the pyridine group is associated with a better adsorption potentiality. In the case of the compounds with a S (BBMS) or an O (BBMO) atom in their structure, and the lower electronegativity of S and higher electron-donating ability over the O atom are associated with a higher corrosion inhibition efficiency.

Hamani et al. [31] associated the corrosion inhibition effect of molecules with adsorption on metallic surface as a result of structural, physicochemical and electronic properties, as the presence of functional groups, such as -N=N-, -CHO, -N=C=N-, R-C=N-R, R=R, and R-OH; molecular area; molecular weight; temperature and electrochemical potential at the metal/solution interface; electronic characteristics of functional groups; steric effect; electronic density of atoms and orbital character of donating electrons. Their assumptions were evaluated in azomethine compounds (synonymous of Schiff bases), all of these exhibited high corrosion inhibition efficiency values (88.3–95.9%) assessed by electrochemical techniques, they demonstrated that the increases on energy of the highest occupied molecular orbital (HOMO), E_HOMO_, which is associated with the electron-donating ability of a molecule, favours the adsorption of corrosion inhibitors on metallic surfaces. Keshavarz et al. [24] ran experiments with 34 corrosion inhibitors from different literature sources to introduce an approach for predicting their corrosion inhibition efficiency. They considered that organic compounds containing functional adsorption centres such as -NH_2_, -OH, -SO_2_-NH-, O and/or N hetero atoms, and aromatic rings have higher corrosion inhibition performance. Molecules such as 1-butyl-2-propylene-2-imidazoline have proven to be highly efficient corrosion inhibitors in HCl media and stainless steel. Likewise, the effect of corrosion inhibition of imidazole (72.6%) in stainless steel is higher than the value reported in the analysis of Bereket, et al. [30]. This behaviour could be associated with differences in the type of metallic surface employed and the analysis conditions. According to the authors, high corrosion inhibition can be the result of (a) covalent interactions between inhibitors and the free metal surface, or (b) electrostatic attraction of the HOMO orbitals of an impregnated metal surface and the lowest unoccupied molecular orbital (LUMO) of a protonated inhibitor.

Most of the corrosion inhibitors studies have been carried out in HCl and H_2_SO_4_ media. Zarrouk et al. [27] mentioned that little work has been done on corrosion inhibition in phosphoric acid solutions. These authors analysed the effect of N-1-napththylethylenediamine dihydrochloride monomethanolate in a 2.0 M H_3_PO_4_ solution. The inhibitor acts as a mixed inhibitor affecting both anodic and cathodic reactions with a negligible shift in corrosion potential as a result of a geometric blocking effect of the adsorbed inhibitive species on the surface of the corroding metal. The displacement in corrosion potential ±85 mV with respect to the corrosion potential (*E_corr_*) of the blank allows to classify a corrosion inhibitor as cathodic or anodic [39]. If the shift in the *E_corr_* values in the inhibited solution is more than 85 mV, the inhibitor can be classified as a cathodic corrosion inhibitor and can be considered a mixed inhibitor if the shift is lower than 85 mV.

The adsorption of this inhibitor is caused by the formation of bonds between the d-orbital of iron atoms and the lone sp^2^ electron pairs on the N-atoms. It was observed that an increase in temperature reduces the corrosion inhibition effect, probably associated with the desorption of inhibitor molecules from the metallic surface. The temperature effect is evident when molecules have attached themselves to a metal by physisorption processes.

Lebrini et al. [23] evaluated the corrosion inhibition effect of two indole derivatives on the corrosion of C38 steel in H_2_SO_4_. During the study, the evaluated compounds were observed to act as mixed inhibitors, these inhibitors are organic compounds with an adsorption centre. From this study analysis, it was possible to hypothesize that an enhanced efficiency may be due to the replacement of a hydrogen atom in pyridine molecule by a methyl group (-CH_3_); such a group has an inductive effect that can be associated with the increase of electron density leading to an enhancement in the inhibition efficiency. The indole derivatives employed by Lebrini and co-workers are polar compounds, thus the electrostatic interaction between the electric field associated with the metal charge and the electric moment of the molecule can contribute to the adsorption process. Taking into consideration the fact that there are protonated species in an acid medium, the interaction presumably occurs between the charged metal and the pyridinium ions.

Khaled [36] employed a series of 14 derivatives of 1,3-pyrimidine reported by Lukovits et al. [7] to construct a mathematical relationship useful to predict the molecular characteristics that define the best corrosion inhibitors. The study also included molecules with a negative “inhibition efficiency”, it mentioned that it is necessary to consider that the inhibition and the activation of the corrosion processes occur simultaneously, and both depend on molecular properties. The analysis included corrosion inhibition values in the interval −28% to 62%, the most negative value (−28%) corresponds to 2-mercapto-4-amino-5-nitro-1,6-dihydropyrimidin. Negative values of corrosion inhibition have been observed also by Lagrenée et al. [40] in the analysis of the same compound at different concentrations. The analysis of data showed that under cathodic and anodic polarisation, this compound increases the corrosion current density, without a noticeable change in the corrosion potential compared to the acidic media. The negative corrosion inhibition values are caused by a catalytic effect, both on the hydrogen reduction rate as well as on the steel dissolution.

The analysis reported by Khaled [36] include information related to frontier orbitals, which is useful in predicting adsorption centres of the inhibition molecules responsible for the interaction with surface metal atoms. It was also observed that the volume of the inhibitor molecule and the molecular orbital energy are important factors correlated with the corrosion inhibition effect. The quantitative structure activity relationship (QSAR) model was built using an important number of molecules described in different projects, such as the analysis performed by Khaled et al. [36], in which the authors included the data of 23 corrosion inhibitors reported in literature (amines, thiourea derivatives and acetylenic alcohols) to estimate its corrosion inhibition on 22% Cr stainless steel. The most effective corrosion inhibitors analysed by Khaled and co-workers [36] included tributylamine, aniline and thiourea derivatives such as 3-dibutylthiourea, 1,3 diethylthiourea and 1,3-dimethylthiourea, the less efficient corrosion inhibitors identified in the analysis were aliphatic amines, isopropylamine, sec-butylamine, propylamine, diethylamine, and n-butylamine; nevertheless, it is worth noting that their corrosion inhibition efficiencies were higher than 60%. Authors have reported that among the most efficient corrosion inhibitors are those compounds with a triple bond [10]. The experimental data of amines, thiourea derivatives, and acetylenic alcohols evaluated by Khaled and co-workers [41] were obtained by Cardoso et al. [29], who tested them as corrosion inhibitors of 13% Cr steel in HCl at 60 °C under experimental conditions designed to avoid complete metal dissolution and to adhere to industrial recommendations. According to the authors, no more than 2% *w*/*v* of the active components are allowed for acidification operations. Formaldehyde was employed to minimize hydrogen penetration. The highest corrosion inhibition efficiency was displayed by tributylamine and aniline compounds (97.8%). Considering that Δ*G_ads_* is an important thermodynamic property, *ln K_ads_* was regarded as a response property to analyze the corrosion inhibition effect.

Imidazoles and benzimidazoles have played a prominent role as inhibitor compounds in the study of corrosion protection [5,34,35]. As stated by Aljourani et al. [5], benzimidazole derivatives interfere neither with metal dissolution reactions nor with proton reduction. These compounds act, rather, as adsorptive inhibitors reducing the anodic dissolution, retarding the hydrogen evolution reaction and blocking the active reaction sites. It has already been mentioned that imidazole and benzimidazole derivatives are not considered to be good corrosion inhibitors; however, differences in their inhibition performance have allowed their comparison. Structural differences such as the alkyl chain length, volume and aromaticity are regarded as critical parameters that affect the corrosion inhibition performance. Gutierrez et al. [34] demonstrated that the presence of substituents as well as their position in imidazole and benzimidazole compounds have a significant effect on the protection of the steel surface. Furthermore, the presence of halogens in the inhibitor structure has been observed to favour the corrosion inhibition efficiency.

Compounds with heteroatoms, including nitrogen, have shown high efficiencies as corrosion inhibitors, which can be attributed to the formation of bonds between the lone pairs of electrons in heteroatoms and the empty orbitals of the metal atoms. The effect of corrosion inhibitors can also be enhanced by other molecular characteristics such as the presence of electrodonating groups. In addition to the molecular structure, the temperature is another factor that can modify the efficiency of inhibition, for example, under certain conditions the temperature favours the diffusion process of the inhibitor to the surface, or it can also be the cause of the desorption of the inhibitor of the metal surface. The environment in which the corrosion process takes place can also be responsible for changes in the efficiency of an inhibitor. The presence of chloride or sulphate ions that can also be adsorbed onto metal decrease the adsorption of corrosion inhibitor. As can be seen in Table 1, there are important differences in the analysis conditions of the corrosion inhibitors. Although an attempt has been made to organize the information in Table 1 from highest to lowest efficiency, the comparison is complex because of the heterogeneity on the experimental conditions.

## 4. S-Containing Corrosion Inhibitors

Many authors have reported the beneficial effect of the sulphur atom in the molecular structure of corrosion inhibitors. Table 2 includes at least four examples of the S-containing corrosion inhibitors evaluated by different groups of researchers. The molecules were ordered from highest to lowest efficiency, considering the best inhibitor in each group. The graphic representation corresponds to the molecules marked with an asterisk.

Lukovits et al. [42] and Khalil [43], employed the same series of thiosemicarbazide and thiosemicarbazone derivatives evaluated experimentally as corrosion inhibitors of mild steel exposed to H_2_SO_4_ to explain the inhibition efficiency in terms of molecular parameters. Khalil explained that derivatives were chosen to alter the electron density of the adsorption centre >C=S. The highest corrosion inhibition efficiency is observed when 1-phenyl thiosemicarbazide is employed. As Khalil stated, the development of the quantitative structure activity relationship can be carried out by means of two different approaches. A first approach (empirical method) where it is considered that each functional group in a molecule contributes in an independent way to the corrosion protection, whereas in the second approach (semi-empirical method), the quantum-chemical properties are associated with the corrosion inhibitor performance.

Amongst the organic sulphur-containing compounds we can include the thiourea derivatives as corrosion inhibitors. Ozcan et al. [44] performed the analysis of thiourea, methylthiourea and phenylthiourea for the corrosion protection of mild steel in H_2_SO_4_, the selected compounds have a similarity: one side of the thiocarbonyl group (-CS-NH_2_). The three evaluated compounds revealed high corrosion inhibition values in the following order: phenylthiourea > methylthiourea > thiourea. The analysis demonstrated that the substitution of the hydrogen atom of the amino group (-NH_2_) by a methyl (-CH_3_) or phenyl group (-C_6_H_5_) causes an increase in the corrosion inhibition efficiency. The inhibition efficiency increase is associated with an increase in the electron density of the functional group caused by the rest of the molecular structure. The thiourea derivatives have been characterized as good corrosion inhibitors as they showed better results than other amine-based inhibitors in acidic media. The thioamide derivatives exist as protonated species in acidic media, the protonation process takes place at the S atom. The protonated inhibitor can be adsorbed electrostatically onto the negatively charged metal surfaces. Through theoretical analysis of thioamide derivatives, it has been demonstrated that the highest value of *E_HOMO_* density can be found in the vicinity of the sulphur atom, meaning that the nucleophilic centre is the sulphur atom, thus giving rise to the formation of a bond which involves the metal and the sulphur atom. This occurs more easily with inhibitors containing S atoms than with those that include N or C atoms in their structure. The S atom in the C=S group has been defined as the most probable centre of adsorption because of its lone pair of electrons [44].

The presence of heteroatoms in molecules favors their corrosion inhibition effect. Actually, more significant increases have been observed in inhibitor molecules including at least two types of heteroatom. N-heterocyclic compounds with N and S heteroatoms in their structure are employed in the protection of steel in acidic environments; examples are thiadiazole, thiazole, and benzothiazole derivatives. 2-Mercaptobenzothiazole is an N- and S-containing heterocyclic compound characterized by its ability to form hydrophobic complexes with metals (e.g., iron, copper, cobalt, and nickel), which is a favorable characteristic in an effective corrosion inhibitor. In the theoretical analysis, 2-mercaptobenzothiazole has been considered to exist as two tautomeric forms: thiole and thione. The analysis of different mercaptobenzothiazole structures has demonstrated that its molecules are completely planar which enables that a strong interaction occurs between the inhibitor molecules and the metal surface [45]. Benali et al. [47] studied the behaviour of 2-mercapto-1-methylimidazole as a corrosion inhibitor of carbon steel in HClO_4_ 1.0 M and, as mentioned before, this molecule can be present in its two tautomeric forms. If nitrogen and sulphur atoms are part of the molecule structure, then it would be possible to predict that this compound can be adsorbed on the metal surface by the interaction of its lone pairs of electrons and such surface, a process enhanced by the low-energy of vacant d orbitals of the iron atoms. The theoretical analysis of the tautomeric forms helped researchers identify that the highest electron density is located in the vicinity of the sulphur atom, favouring the formation of a bond between the metal and the S atoms over the N or C atoms.

Semicarbazones and thiosemicarbazones have been used as corrosion inhibitors because they have the ability to form complexes with various metals. Their corrosion inhibition effect can be attributed to the nitrogen and sulphur atoms in their molecular structure. It has been mentioned that sulphur-containing compounds are more effective in corrosion protection than similar oxygen-containing compounds, thus it is possible to consider that thiosemicarbazones are more efficient than the analogous semicarbazones. Goulart, et al. [46] worked with semicarbazones and thiosemicarbazones (all containing azomethine group -CH=N- and π electrons) in the corrosion protection of carbon steel AISI 1020 (American Iron and Steel Institute) exposed to HCl 1.0 M, these compounds promote a decrease in both the anodic and cathodic current densities and a shift in the corrosion potential towards the anodic direction; such behaviour has been associated with mixed inhibitors. These compounds cause an effect on the reduction of the anodic dissolution of steel and slow the cathodic evolution of hydrogen when they are added to acidic media. This analysis demonstrated that the adsorption centres in the thiosemicarbazones correspond to the azomethine group, the nitrogen atom of the NH group, and mainly the sulphur atom, whereas in the semicarbazone compounds the adsorption centres are only the azomethine group and the nitrogen atom of the NH group. The effectiveness of the thiosemicarbazones selected by Goulart and co-workers is also related to the electron density increased by the electron-donating groups (-OCH_2_CH_3_, -OH) located at the para position of the benzaldehyde ring; the electron-donating groups (such as -OCH_3_) found at the meta position do not lead to a significant increase in the inhibition efficiency because the meta position cannot contribute to the electron density by resonance. The difference between thiosemicarbazones with a thiocarbonyl group (C=S) and semicarbazones with a carbonyl group (C=O) is the greater polarizability of the sulphur atom of the former, which enhances its adsorption processes.

As can be seen in Table 2, molecules with sulfur atoms in their structure have high corrosion inhibition efficiencies. The sulfur atom in a corrosion inhibitor represents a potential centre for adsorption on the surface to be protected. Furthermore, the efficiency of inhibitors with a sulfur heteroatom can be modified according to the rest of the molecular structure. Good corrosion inhibitors have high electron densities in the vicinity of the sulfur atom. Another factor also attributed to the corrosion inhibition efficiency is the planarity of the molecules, since it has been mentioned that this characteristic favors the interactions between the inhibitor molecules and the metal surface.

## 5. O-Containing Corrosion Inhibitors

Many authors have studied the effect of the oxygen atom in the molecular structure of corrosion inhibitors. Table 3 includes examples of molecules that have been analyzed to observe the effect of oxygen on corrosion inhibition efficiency. The experimental conditions and efficiencies obtained using different techniques are also included.

Indanone is a class of ketone; a bicyclic compound that consists of a benzene ring fused with a cyclopentanone ring. Due to their biological activity, many derivatives of indanone have been studied as antiviral and antibacterial agents, anticancer drugs, pharmaceuticals in Alzheimer’s treatment, cardiovascular drugs, insecticides, fungicides, herbicides, and drugs for hepatitis C treatment. Indanone derivatives have been also investigated as corrosion inhibitors for mild steel in hydrochloric acid. Saady et al. [21] used three indanone derivatives as corrosion inhibitors reaching corrosion inhibition efficiencies in the interval 87–92% (evaluated by weight loss). The selected indanone derivatives are considered environmentally benign, their effect as corrosion inhibitors can be attributed to the aromatic rings with conjugated double bonds that allow their easy adsorption on metallic surfaces, as well as to the presence of an electron-releasing methyl group that enhances the adsorption of the molecule on a metallic surface. The carboxyl groups of these compounds facilitate their solubility in acidic media. It was observed that the substitution of the hydroxyl group by a methyl group and a phenyl ring, or even only phenyl group would improve the inhibition performance. The indanone compounds were defined as mixed-type inhibitors whose inhibition effect might be reduced by increasing the temperature.

Authors such as Baharami et al. [48] studied the relationship between the molecular structure of organic compounds and their inhibition efficiency by means of quantum chemical calculations. According to the authors, 1-((4-chlorophenyl)(2-hydroxynaphtalen-1 yl)(phenyl)methyl)urea could be considered commercially important considering its effect as corrosion inhibitor even at low concentrations (8–10 ppm range). Through Fourier transform infrared (FTIR) analysis, it was possible to observe that the oxygen atom and carbonyl group did not contribute to the adsorption process, whereas the N and O from N–H and O–H could serve as active centres for adsorption.

The research on hydantoin derivatives by Olasunkanmi et al. [13] confirmed that the inhibitive potential can be attributed to the presence of oxygen and nitrogen atoms as adsorption sites for molecular interaction. The methyl and Br substituents in the evaluated compounds contributed to their electron-donating and electron-withdrawing abilities, which facilitate the donor–acceptor interaction with steel. These compounds are adsorbed on the steel surface and block the active sites without changing the corrosion mechanism.

The O-containing corrosion inhibitors shown in Table 3 show high inhibition efficiency values, which are the result of the set of characteristics in the molecular structure, such as the presence of π electrons of the aromatic rings, the lone pair electrons of the atom oxygen, and functional groups such as carboxyl. The corrosion inhibition values of some compounds are associated with the inductive effects caused by the presence of atoms or functional groups in different positions of the molecule. In studies carried out by different researchers, it has been shown that in some molecules the presence of methyl groups increase their ability as an electron donor species and therefore improves their inhibition efficiency.

## 6. Multi-Heteroatom-Containing Corrosion Inhibitors

For the selection of high-performance corrosion inhibitors, some researchers tend to choose molecules with a high number of heteroatoms (Table 4), or molecules featuring the characteristics of better corrosion inhibitors. However, it has been found that some heteroatoms work more efficiently as adsorption centres than others, or that the presence of certain substituents increases the electron density of the functional groups leading to stronger interaction between the corrosion inhibitor and the metallic surface.

Lebrini et al. [18] tested macrocyclic polyether compounds (containing a 1,3,4-thiadiazole moiety) as corrosion inhibitors for mild steel exposed to HCl media as there were reports about the ability of this type of compounds to interact with metallic surfaces. The researchers observed that there is an enhancement in the inhibition corrosion efficiency as a result of the extent of the polyethylene glycol unit. The number of oxygen atoms in the polyether ring increases the adsorption strength through the non-bonding electron pairs and the vacant orbitals of the metal surface. In the evaluated compounds, the increase in the molecular area promoted an increase in the corrosion inhibition efficiency since the compounds had a greater adsorption ability [18].

The formation of the adsorption film on mild steel has been studied using techniques like scanning electron microscopy (SEM), energy-dispersive X-ray spectroscopy (EDS), and FTIR, whereas quantum chemical calculus and QSAR have permitted the identification of the best reactive sites in a molecule to interact with a metallic surface. Muralana et al. [4], tested five sulphonamides molecules that are classified as green corrosion inhibitors, the researchers found that the preferred sites for interaction with mild steel were located in the aromatic rings and in the heteroatoms. The FTIR spectra allowed it to be determined that the most characteristic absorption bands disappeared, among them the SO_2_ signal (possible electron donor), concluding that the O atom of the SO_2_ functional group might be the preferred site for interaction between the d orbitals of steel and the sulphonamides.

The theoretical report of Shojaie et al. [50] indicated that the molecules with N atoms are preferentially protonated in acidic medium, contrary to the behaviour of compounds with S and O atoms. The researchers conducted the analysis of two pyrimidine derivatives (S and N compounds) and observed that there was a correlation between the molecular mass and the inhibition efficiency; the adsorption on the metal surface improved as the molecular mass increased. They associated a higher number of heteroatoms in a molecule with its superior interaction capacity with a metallic surface. Atoms with the highest negative partial atomic charge interact more strongly with a metallic surface through a donor–acceptor interaction [50]. Caliskan et al. [14] noticed in their experimental study of pyrimidine derivatives that these compounds work as mixed-type inhibitors, both cathodic and anodic reactions were influenced by the presence of compounds in the corrosive medium, being the anodic effect the most significant behaviour. The greater inhibition effect achieved by pyrimidine derivatives is attributed to the sulphur atom.

The current need for environmental protection has prompted the analysis of non-toxic and eco-friendly molecules, such as amino acids, as corrosion inhibitors. Compounds such as L-cysteine, L-leucine, L-alanine, and glycine have been evaluated as mild steel corrosion inhibitors reaching efficiencies in the interval 56–82%. According to the information obtained Eddy et al. [51] through FTIR, there are bonds in every compound that enable its adsorption on the metallic surface, N-O and C-N are associated with L-cysteine adsorption; C–N, C–O, N–O, and N–H are considered to be responsible for glycine adsorption; C–N and N–O help L-leucine adsorb on the analyzed surface, and finally, C–N, N–O, O–H, and C–H are bonds that can be associated to L-Alanine adsorption. Their theoretical analysis demonstrated that the most negative charge in the studied amino acids corresponds to oxygen atoms; however, as nitrogen is more electronegative than oxygen, this site is considered to be the preferred site for electrophilic attack. The evaluated compounds donate electrons to the metal and accept the lone pair of electrons from Fe, generating a feedback bond that can be characterized by different theoretical descriptors such as *E_HOMO_*, *E_LUMO_* and experimental data of corrosion inhibition. The authors mentioned that the corrosion inhibition processes can be evaluated as a composite function of some quantum-chemical descriptors [51].

Aouniti et al. [10] also conducted a research project on amino acids used for the corrosion protection of Armco Iron in 1.0 M HCl. Experimental data allowed sulphur-containing inhibitors and sulphur-free amino acids to be compared. It was demonstrated that those compounds that possess a sulphur atom in their molecular structure make the best inhibitors. All the inhibitors investigated had an acidic and amine functions in the molecular structure (R-COOH-NH_3_^+^). According to results, the variation in inhibition efficiency is a result in the change of the substituents located at the end of the radical (R), which can influence the number of adsorption active centres in the molecule and their charge density, molecular size, adsorption mode and formation of metallic complexes. Aouniti and co-workers mentioned that inhibition efficiency changes with the number of CH_2_ groups in the radical (R), as follows:

- If the substituent at the end of R is an electron-donating substituent, the inhibition efficiency rises as the number of CH_2_ increases.
- If the substituent is an electron acceptor, the inhibition efficiency decreases as the number of CH_2_ increases.

Eddy et al. [22] carried out the study of carbazones (N and S containing inhibitors) as corrosion inhibitors for mild steel exposed to 0.1 M HCl. Unlike other studies, long times of exposure to the corrosive medium were used in this gravimetric analysis. The corrosion product was removed every 24 h using a solution of 50% NaOH and 100 g⋅L^−1^ of zinc dust. The results reported a change in weight over a period of 168 h. The effectiveness of some carbazones as corrosion inhibitors is associated with a chemical adsorption process because the inhibition efficiency was found to rise after increasing the temperature. The corrosion inhibition efficiencies obtained by weight loss were within an interval of 36–81%.

Large organic molecules with a significant number of heteroatoms have been proposed as efficient corrosion inhibitors, because some of these heteroatoms can act as preferential adsorption centres and because of the molecular mass has been related to the inhibition efficiency. Larger molecules have been reported to block metal surfaces more efficiently, reducing the contact with the corrosive medium, however, in many cases it is important to take into account factors such as the planarity of the molecules and the roughness of the surfaces that can also affect inhibitor-surface interactions.

## 7. Molecular Descriptors Associated to Corrosion Inhibitors

There is a growing interest in the development of quantum-chemical studies because the inhibition activity of molecules can be associated with certain theoretical parameters, such as the highest occupied molecular orbital energy (*E_HOMO_*), the lowest unoccupied molecular orbital (*E_LUMO_*), dipole moment and Mulliken atomic charges. The quantum parameters of organic inhibitors can be used to study their interaction with a metallic surface. Molecular dynamics simulation is helpful to study the adsorption of inhibitors on a metallic surface because it provides molecular-level information on the way the inhibitor is adsorbed on the metal. Theoretical and computational chemistry is a useful, powerful tool to choose the most appropriate inhibitor by understanding the inhibition mechanism before performing any experiment [52]. QSAR applied to corrosion analysis is a theoretical method convenient to correlate certain molecular structural parameters and the corrosion inhibition efficiency of a group of compounds with similar characteristics; it is a model of pattern recognition that can be implemented to define a trend in the corrosion efficiency scanning the variations in the inhibitor structural parameters. The QSAR approach was initially used in pharmacology in 1964 because Hansch and Leo observed that the variation of pharmacological efficiency of drugs might be explained in terms of different variables related to the molecular substituents. One of the main objectives of QSAR studies is to reduce the cost of research [42]. QSAR can be advantageous in the identification of compounds with high yields and characteristics that might be desirable from an environmental point of view. It is recommended to include a large number of compounds with similar backbones to enhance the QSAR accuracy; however, as stated by Mousavi and co-workers, that is in itself a disadvantage of this model since it might be difficult to find a large number of compounds with similar backbones and their corrosion inhibition experimental data obtained under the same conditions [52]. Some authors have mentioned that the cluster model based on density-functional theory (DFT) calculations is a suitable tool for modelling the inhibitor–surface interaction mechanism and for describing the effect of the inhibitor structural nature and the metal of interest. This method considers factors such as, inhibitor molecule, metal, and solvent which are ignored by the QSAR analysis. The cluster model evaluates the inhibitor–surface interaction energy vs. the experimental corrosion inhibition. The main advantage of the DFT calculation is to help researchers theoretically identify corrosion inhibitors that exhibit low interaction energies and discard them from the investigation, whereas the objective of QSAR is fundamentally based on two questions: (1) what structural and electronic properties of a molecule determine its activity, and (2) what structural and electronic properties can be altered to improve such activity [36].

As mentioned, the development of mathematical models (linear and non-linear) is possible through the analysis of quantum-chemical parameters and experimental data obtained from compounds with similar characteristics. Table 5 includes some examples of typical descriptors studied to understand the characteristic molecular factors of a good corrosion inhibitor.

The DFT model based on the Hohnenber–Kohn theorems is a significant tool for modelling and developing studies on chemical reactivity. It is based on the principle that the energy of a molecule can be determined by its electron density [51].

The theory on frontier orbitals is useful to predict adsorption centres in the inhibitor molecule where there would be a higher possibility for interaction with the metallic surface. The E_HOMO_ is a descriptor commonly used in the correlation analysis of organic compounds and their corrosion inhibition capacity. High values of E_HOMO_ indicate the tendency of a molecule to donate electrons to appropriate acceptors that possess low energy and empty molecular orbitals. The analysis of inhibitors has demonstrated that molecules with high E_HOMO_ are associated with stronger chemisorption and greater inhibition efficiencies, whereas some researchers associate a negatives E_HOMO_ coefficient to physical adsorption [47,48]. The theoretical analysis of E_HOMO_ in molecules with pyrimidine rings has demonstrated that the most suitable sites for an electrophilic attack are located in the surroundings of nitrogen atoms [36]. The analysis of E_LUMO_ density provides information on the nucleophilic sites [4]. Good corrosion inhibitors are characterized by having low values of E_LUMO_ [48]. The best corrosion inhibitors are normally organic molecules that easily offer their electrons to the unoccupied orbital of the metallic surface to form a coordinate covalent bond and which may also accept free electrons from the metal; different trends have been observed by researchers.

The energy difference (ΔE) is a measure for the stability of the complex formed on the metallic surface. The analysis of Gao et al. [54] showed that a decrease in ΔE is associated with the high quality of the formed film, which is a result of a substantial accumulation of corrosion inhibitor on the metal. The dipole moment (μ_D_) like the energy difference is useful to provide information about the electronic interaction of the corrosion inhibitor with the metal. According to Gao and co-workers, the dipole moment is related to the hydrophobic character of molecules. At lower values of μ_D_, the accumulation of inhibitor is higher enhancing the corrosion inhibition effect; however, the analysis of the relationship between the dipole moment and the inhibition efficiency has yielded confusing results as both positive and negative correlations have been reported [47,51,54,59,60].

The ΔE is also related to the hardness and softness of a molecule. A soft molecule (low ΔE) is expected to be more reactive than a hard molecule [51]. Those molecules with a small ΔE have shown greater reactivity than molecules with a large ΔE value [4]. Low ΔE values are linked with good inhibition efficiencies because the energy required to remove an electron from the last occupied orbital is also low [61].

The electron affinity (*A*) and ionization potential (*I*) are related to the orbital molecular energies [12] as explained by Koopman’s theorem:

(3) $$I=-E_{HOMO}$$

(4) $$A=-E_{LUMO}$$

Vosta et al. [62] stated that there is an important correlation between the inhibition efficiency and the *E_HOMO_*, and the *E_LUMO_*. Their results showed that the inhibition efficiency increases with the decrease of the ionization potential, as they mentioned, the least firmly bonded electrons in the limit HOMO orbital are those richest in energy. If the ionization of a molecule is somehow favoured, the molecule acts as an electron donor to block the corrosion reaction. The electron affinity analysis has shown that effective inhibitors are bad electron acceptors. Substances which are prone to reduction may behave as corrosion stimulators [62].

The electronic hardness (*η*) and softness (*S*) are quantities introduced by Pearson; in the early stage of the reactivity theory, these are part of the hard-soft-acid-base principle (HSAB). According to Pearson, the species are soft if their valence electrons are easy to polarise or to remove while otherwise are considered hard species [36]. According to the HSAB principle, an inhibitor works as a Lewis base, whereas the metal behaves as a Lewis acid. Lewis bases are nucleophiles that reduce agents by donating electrons, whereas acids defined by Lewis are electrophiles that other oxidize substances by accepting their electrons [63]. In a system with certain number of electrons (*N*) at a fixed external potential and total energy (*E*), the electronegativity (*χ*) and hardness (*η*) are defined by the first and second derivatives of energy,

(5) $$\chi =-\mu =-{\left(\frac{\partial E}{\partial N}\right)}_{\nu \left(r\right)}$$

(6) $$\eta ={\left(\frac{\partial^{2}E}{\partial N^{2}}\right)}_{\nu \left(r\right)}$$

where *ν(r)* and μ are the external and electronic chemical potentials, respectively.

The electronegativity (*χ*) is considered the power of an electron or group of atoms to attract electrons toward itself, and the hardness measures the resistance of an atom to a charge transfer, both values can be calculated from the *E_HOMO_* and *E_LUMO_*, according to the following equations [57,64]:

(7) χ=−12(EHOMO+ELUMO)

(8) η=−12(EHOMO−ELUMO)

The ionization potential (*IP*) and electronic affinity (*A*) of the inhibitors can be calculated by means of the equations below, taking the values of the electronic energy into consideration Equations (9) and (10) [25]:

(9) $$IP=E_{\left(N-1\right)}-E_{N}$$

(10) $$A=E_{N}-E_{\left(N+1\right)}$$

where *E*_(*N* − 1)_, *E_(N)_*, and *E*_(*N* + 1)_ correspond to the ground state energies of the system with *N* − 1, *N* and *N* + 1 electrons. The electronegativity, hardness, and softness are related to electron affinity and ionization potential according to the next equations [12]:

(11) χ=(IP+A)/2

(12) η=(IP−A)/2

(13) σ=1/χ=2/(IP−A)

It has been described that in a Fe-inhibitor system, the electrons flow from lower *χ* (inhibitor) to higher *χ* (Fe) until the chemical potentials become equal. The descriptor associated with the fraction of transferred electrons (Δ*N*) is discussed in several reports. The Δ*N* and the initial molecule–metal interaction energy (Δ*ψ*) can be calculated according to Equations (14) and (15), respectively) [25]:

(14) $$\Delta N=\frac{\left(\chi_{Fe}-\chi_{inh}\right)}{2\left(\eta_{Fe}-\eta_{inh}\right)}$$

(15) $$\Delta \psi =-\frac{{\left(\chi_{Fe}-\chi_{inh}\right)}^{2}}{4\left(\eta_{Fe}+\eta_{inh}\right)}$$

where *χ_Fe_* and *χ_inh_* are the electronegativity values of iron and the corrosion inhibitor, respectively, *η*_Fe_ and *η*_inh_ denote the hardness of iron and the corrosion inhibitor, respectively.

The theoretical values of *χ_Fe_* and *η_Fe_*, are 7 eV⋅mol^−1^ and 0 eV⋅mol^−1^, considering the metallic surface where iron is the major constituent [50,65]. According to this descriptor, the transference process is associated to Δ*N* values, when Δ*N* > 0 the electron transference occurs from the inhibitor compound to the metallic surface. Δ*N* < 0 is characteristically observed in the inverse processes [12]. Lukovits et al. [7], mentions that the precise term to refer to Δ*N* is “electron-donating ability”, which does not imply that this descriptor literally represents the number of electrons leaving the donor and entering the acceptor molecule. In the analysis carried out to associate the electronic structure and the corrosion inhibition efficiency, the researchers observed that when Δ*N* < 3.6, the inhibition efficiency increased as the values of Δ*N* became greater. However, the inhibition decreased when Δ*N* > 3.6, this behaviour was associated with inhibitors that may have become attached to some electron acceptor species in the solution before they could form a bond on the metal/metal oxide surface, which led to a less efficient corrosion inhibition.

The electrophilicity index (*ω*) is a chemical reactivity parameter; this descriptor is a quantitative measure of the global electrophilic power of a molecule. The electrophilicity index was proposed as a measure of energy lowering caused by the maximal electron flow between a donor and an acceptor, ω is calculated as follows [57,66]:

(16) $$\omega =\frac{\chi^{2}}{2\eta }$$

An approximate electrophilicity value may be calculated through the following equation [58]:

(17) $$\omega \approx \frac{{\left(I+A\right)}^{2}}{8\left(I-A\right)}$$

The electrophilicity index is associated with the stabilization energy gained by a molecular system upon receiving an electron from the environment, the high or low values of this descriptor can explain the good electrophilicity or nucleophilicity of a molecule. Good corrosion inhibitors generally have a high nucleophilicity degree [67].

Two global indicators of chemical reactivity were proposed by Gazquez et al. [58] which are useful to calculate the electrodonating (*ω*^−^) and electroaccepting (*ω*^+^) powers, such descriptors indicate the capability of a molecule to donate and accept a small fractional amount of charge and can be are calculated by the equations below [58,68]:

(18) $$\omega^{-}=\frac{{\left(3I+A\right)}^{2}}{16\left(I-A\right)}$$

(19) $$\omega^{+}=\frac{{\left(I+3A\right)}^{2}}{16\left(I-A\right)}$$

As mentioned, it has been reported that if Δ*N* is smaller than 3.6 eV, the inhibition efficiency increases while raising the value of *ω*^−^ at the metallic surface interface [50]. The theoretical analysis completed by Camacho-Mendoza et al. [35] to evaluate the relationship between the structure and corrosion inhibition of N-containing inhibitors showed that *ω*^−^ is a descriptor correlated with the inhibition efficiency process.

While developing computational details, some authors conduct the study of an aqueous environment using the self-consistent reaction field theory based on Tomasi’s polarized continuum model (PCM). The PCM calculations has allowed them to observe that the relative energies of the compounds diminish as the polarity of the solvent and the size of the molecule increase. When the solvation of the inhibitor molecule takes place, some changes in the charge distribution occur producing a larger dipole moment than that obtained through the gas-phase analysis. An additional way to obtain information about the distribution of electrons is to study the polarizability (α) of the system. Polarizability is another important property used to visualize the hard-soft concept; it is a measure of the linear response of the electronic cloud of a chemical species to a weak external electric field. α is the dipole polarizability explained by the equation: $\alpha =\left(\alpha_{xx}+\alpha_{yy}+\alpha_{zz}\right)/3$.

There is an inverse relationship, η∝1α1/3 between hardness and polarizability. A minimum polarizability principle has been established, “the natural evolution direction of any system is towards a state of minimum polarizability”. A state of minimum polarizability usually can be associated with higher stability [63,69,70]. Higher values of α have been related to strong adsorption process and a high inhibition efficiency. A high correlation between the corrosion inhibition efficiencies and molecular polarizability was found (96%) [25,55].

The Fukui functions are useful in the analysis of the response of the whole molecular spectrum, not only the frontier orbitals. The study of the Fukui indices for each of the atoms in a molecule and the analysis of the global hardness help define a more complete scheme of its reactivity. The local reactivity is investigated by means of the condensed Fukui function taking a look at each part of the molecule on the basis of the chemical behaviour associated with the different functional groups. The most susceptible site to nucleophilic attack is the place where *f_k_^+^* displays its maximum value, whereas the best site for electrophilic attack is defined by the maximum value of *f_k_^−^*. From the analysis of pyrimidine derivatives, it is possible to deduce that if the *f_k_^−^* is only taken into consideration for nitrogen, oxygen, sulphur, and carbon atoms, an increased value would be predictable for a high inhibition efficiency of the compounds. Therefore, it can be concluded that compounds with more electrophilic character have various active centres to get adsorbed on the iron surface. The simplified form of the Fukui functions can be written as [36,45]:

(20) $$f_{k}^{+}=\left[q_{k}\left(N+1\right)-q_{k}\left(N\right)\right]$$

(21) $$f_{k}^{-}=\left[q_{k}\left(N\right)-q_{k}\left(N-1\right)\right]$$

where *q_k_(N)*, *q_k_*(*N* + 1), and *q_k_*(*N* − 1) are the electron population of the kth atom in the *N*, *N* + 1 and *N* − 1 electron systems, respectively.

Local softness (*s*) can also be expressed as the product of the Fukui functions and the global softness (σ) according to the following equations [22,53]:

(22) $$s^{+}=\left(f^{+}\right)\sigma$$

(23) $$s^{-}=\left(f^{-}\right)\sigma$$

The higher the values of s^+^ are, the higher the nucleophilicity of the molecule is.

Among the most useful calculations reported in literature is the analysis of descriptors in presence of the solvent effect. A lower solvation energy is a signal of an easier inhibitor adsorption on the metal surface, which, of course, would mean achieving a high inhibition efficiency [53]. Another characteristic that has been analysed because of its effect on corrosion inhibition is the hydrophobicity, determined by *Log P* (partition coefficient). Higher *log P* values are associated with lower solubility. It is believed that hydrophobicity alters the oxide/hydroxide layer on the metal surface modifying the corrosion inhibition process [25]. Zhao et al. [71] investigated the protection of mild steel using phthalocyanines as corrosion inhibitors and sulfonation as a method to improve the solubility of molecules. Sulphonation was also identified to be responsible for a change in the dipole moment (from 3 to 23) and decreasing *log P* from 6 to about 2. The researchers reported that an increase in solubility can make the inhibitor more effective, and that increase in the dipole moment enhances the physical adsorption of the inhibitor improving the inhibition effect as well. The study of corrosion protection mechanisms of oleic imidazoline by Ramachandran et al. [72] demonstrated that the formation of the self-assembled monolayer is dependent on having a partition coefficient bellow the critical value so the monolayer responsible for Fe protection can be rapidly formed. Inhibitors with sufficient solubility and transport rate provide fast protection against corrosion.

The analysis of Mulliken charges is useful to identify heteroatoms with negative charges that can be potential binding sites in the corrosion protection processes. It is known that a centre with a higher negative charge may be willing to give its electronic charge to the empty orbital of a metallic surface. From Mulliken charges, it is possible to obtain the total negative charge (TNC) of a corrosion inhibitor, high values of this parameter are associated with high adsorption on a metallic surface [67].

Keshavarz et al. [24] worked in the generation of QSAR models to predict and evaluate some organic compounds as corrosion inhibitors. They selected molecular characteristics as alternative descriptors such as the number of nitrogen atoms n(N), the sum of the number oxygen atoms and amino groups *n(O+NH_2_)*, and the structural *η*^+^ and *η*^−^ parameters that affect positively or negatively, respectively). This implied that some specific functional groups and molecular moieties could be used to adjust the effects on n(N) and *n(O+NH_2_)*. For example, the parameter *η*^+^ can be related to the presence of electron-donor groups which increase the electron density, and *η*^−^ can be associated with electron-acceptor groups which reduce the electron density of molecules.

As explained throughout this section, the activity of a molecule as a corrosion inhibitor can be attributed to various parameters that can be theoretically analysed. The compression of the inhibitor–metal surface interaction allows the selection of molecules whose characteristics favour the efficiency of corrosion inhibition. For example, the analysis of the energies of the molecular orbitals allows probable sites for electrophilic attacks to be identified, it also allows obtaining information about the nucleophilic sites and, based on this knowledge, species can be proposed as good or bad corrosion inhibitors. Energy analysis is also useful to determine the stability of complexes formed on the metal surface. Properties such as the reactivity of a molecule make it possible to predict the ease with which it will interact with metal atoms. The simultaneous analysis of different descriptors allows improving the level of prediction about good corrosion inhibitors, which is why different groups of researchers have worked on the generation of equations that involve different molecular characteristics.

## 8. Mathematical Models Applied to Predict the Corrosion Inhibition Efficiency

Corrosion is responsible for significant economic waste and is deemed a risk factor in industrialized countries. This is one of the reasons why fast analysis techniques are an attractive alternative to study corrosion inhibition. Several researchers have demonstrated that some organic compounds with certain general characteristics such as, heteroatoms and aromatic rings, can achieve good results when they are employed as corrosion inhibitors, especially in acidic media.

Novel methods of analysis are valuable tools in the research for more effective corrosion inhibitors as they help avoid the use of typical compounds that pose health and environmental risks, such as corrosion inhibitors containing tin, chromium, and lead [73].

Computational analysis and experimental techniques such as electrochemical impedance spectroscopy, potentiodynamic polarization, and weight loss, have been used by researchers for the generation of mathematical models that allow to define those molecular features associated with good protectors against corrosion. Computational techniques are also useful to analyse the mechanisms of action of corrosion inhibitors. Researchers usually take advantage of experimental databases of compounds previously tested as corrosion inhibitors in different media and for several metals with the aim of generating predictive models and reducing costs and time invested in analysis.

Among the objectives of the construction of mathematical models are (a) the understanding of the action mechanisms of the evaluated compounds and, (b) the design of new corrosion inhibitors with desirable properties for metal protection.

It should also be considered that some changes in theoretical studies can be observed in descriptors depending on the computational analysis conditions taken into account, for example, the phase specified in the analysis, which might be either gas or aqueous. Such changes can clearly be noticed in the obtained determination coefficients. Authors such as Aboelnga et al. [53], in the study of triazoles and mercaptobenzothiazoles used as corrosion inhibitors demonstated differences in theoretical studies as a result of selected phase

Many attempts have been made to explain variation of the relationship between structural parameters and corrosion inhibition efficiency (*IE*) by means of the equation below:

(24) IE=Ax+By+k

where *x* and *y* correspond to structural (or electronic) parameters of the inhibitor. The variables *A, B,* and *k* are the regression coefficients to be optimized. The lack of adjustment can be resolved using multiple linear regression approaches:

(25) $$IE_{exp}=Ax+Bx^{2}+k$$

Eddy et al. [22], as other authors, have tried to correlate some quantum chemical parameters with experimental corrosion inhibition efficiencies using multiple linear regressions; however, it has not been easy to find a simple or direct linear relationship. Linear models approximate a calculated corrosion inhibition efficiency, *IE* (%) through certain quantum chemical parameters according to the equation:

(26) $$IE=Ax_{j}C_{i}+B$$

where *A* and *B* are constants obtained by regression analysis; *x_j_* is a quantum chemical index which is characteristic of molecule *j*; *C_i_* is the concentration of the inhibitor. Nevertheless, the analysis revealed that the inhibitor efficiency depends on composite functions that cannot be expressed by linear models. This made the non-linear model proposed by Lukovits et al. [42] a common approach to study the interaction of corrosion inhibitors with a metallic surface. This model considers that the concentration of inhibitors affects the corrosion inhibition results, and it is based on the Langmuir adsorption isotherm, where it is assumed that the surface coverage (θ) is equivalent to *IE.*

(27) $$IE=\frac{\left(Ax_{j}+B\right)C_{i}}{1+\left(Ax_{j}+B\right)C_{i}}$$

Table 6 includes some examples of mathematical models generated to analyse the corrosion inhibition efficiency of different organic molecules and their corresponding determination coefficients (*R*^2^).

Ashassi-Sorkhabi et al. [3] carried out a QSAR analysis to study the effect of molecular structure of three Schiff base compounds obtaining theoretical data using the AM1 semi-empirical method. The authors found an excellent correlation between the theoretical corrosion inhibition efficiency and the quantum-chemical parameters (*E_HOMO_*, *E_LUMO_*, and µ_D_) using linear and non-linear models. According to the linear mathematical model obtained, the negative coefficient of *E_LUMO_* proves that the d orbitals of steel accept electrons from the employed compounds and feedback bonds form between steel and inhibitors which increase the chemical adsorption and, thus, the corrosion inhibition.

Other authors have performed analyses with unconventional descriptors. Cardoso et al. [29] worked with the AM1 method for the analysis of descriptors from 23 compounds (amines, thiourea, derivatives and acetylenic alcohols). Such descriptors were correlated with weight loss using three different statistical methodologies: ordinary least squares (OLS) with simple descriptor selection based on R^2^ values, second-order cross-validation OLS procedure (SOCV-OLS), and a third method based on regular partial least squares (PLS). Their calculations were based on the Langmuir adsorption function (*ln K_ads_*) and took the following non-common descriptors into consideration: number of RNH_2_ groups (A_1_), R_1_R_2_NH groups (A_2_), R_1_R_2_R_3_N groups (A_3_), phenyl groups (NB), number of cyclic carbon rings (NC), number of CS bonds (NCS), number of triple CC bonds (NT), number of OH groups (NOH), average number of carbon atoms (NCR), branching number (NR), and number of moles of inhibitor (N), dimerization energy (ED), molecular mass (M), polarizability (P), charges in different atoms and polar groups (C, C_1_, C_2_, C_12_, C_13_, C_14_) and volume (V). When 15 descriptors were considered in the OLS, the model obtained had a high regression coefficient *R*^2^ of 0.979. Common descriptors calculated by OLS and SOCV-OLS included polarizability, charge between two neighbouring groups (C_12_) and the number of OH groups. It was possible to conclude from the PLS analysis that the most significant attachment occurred via secondary amines, and that there is an important number of insignificant descriptors in the model.

Lukovits et al. [42] performed a QSER (quantitative structure-efficiency relationships) analysis of thiosemicarbazides and thiosemicarbazones using *ln K* as the dependent variable of the analysis. According to the authors, the use of *ln K* instead of *K* allowed treating the variation of inhibition efficiency in terms of linear free-energy relationships through a non-linear regression technique. In the generated models, the variation of inhibition efficiency can be explained in terms of *E_HOMO_* and *μ*_D_, or alternatively employing ΔE as can be observed in Table 6. It was observed that increasing values of *μ*_D_ cause a decrease in the inhibition efficiency, which allowed the researchers to associate fewer polar molecules with higher inhibition efficiencies. The authors found that quantum-chemical indices as *E_LUMO_* and atomic charges are less efficient in the explanation of the variation of the corrosion inhibition efficiency.

Guo et al. [25] investigated the corrosion inhibitive performance of three triazole derivatives during the acidic corrosion of a mild steel surface using DFT with hybrid B3LYP functional considered as the most popular and accurate one for the analysis of organic molecules in combination with the 6-31G basis. These authors described the aqueous environment through the self-consistent reaction field theory based on Tomasi’s PCM. They observed that the relative energies of the compounds are lower when the solvent polarity and the molecule size increase. The solvation of corrosion inhibitors is responsible for significant changes in the charge distribution resulting in a large dipole moment compared with that obtained in the gas-phase calculation. They observed that the polarizability values of molecules increased in presence of water favouring the adsorption process. The inhibition efficiency of the three corrosion inhibitors evaluated rose with the increase of *log P*. The authors presented 10 mathematical models based on the Langmuir isotherm (in aqueous and gas phase), in which they combined three of the descriptors, *E_HOMO_*, *E_LUMO_*, *μ*, *V*, ΔE, *log P* and *α* obtaining correlation coefficients higher than 0.9, two mathematical models are displayed in Table 6.

Bereket et al. [30] studied 11 imidazole derivatives as corrosion inhibitors; the group of authors divided the compounds into three series according to their behaviours to investigate the relation between structure and inhibition efficiencies. The best inhibition efficiency correlation was obtained vs. total charges (R^2^ = 0.8691) in aqueous phase. The result was associated with the effect of dihedral angles, which demonstrated that the molecules are almost planar. The correlation vs. *E_HOMO_*, *E_LUMO_*, and ΔE reached correlation coefficients greater than 60% which are considered acceptable in the quantum-chemical calculations of corrosion analysis. Considering that the chemisorption of organic molecules is associated with *E_HOMO_* and *E_LUMO_* (descriptors involved in the formation of feedback bonds), a regression analysis of the inhibition efficiency vs. these descriptors was performed for a series of compounds; high values of R^2^ were obtained (0.82–0.99).

Keshavarz et al. [24] used a simple approach to predict the corrosion inhibition of imidazole, benzimidazole derivatives and linear organic compounds that contain several polar functional groups. Researchers considered that QSAR models are based on complex molecular descriptors that require many computational resources and expert analysts, and this encouraged them to construct a mathematical model based on the molecular structure of the compounds, which can be easily calculated. Their mathematical model (obtained by multiple linear regression method) is included in Table 6; the variables involved were the number of nitrogen atoms *n(N*), the total number of oxygen atoms and amino groups *n(O+NH_2_)*, *η*^+^ and *η*^−^ that correspond to the positive and negative effects of structural parameters on the inhibition efficiency and which also can be used to adjust the effects of *n(N)* and *n(O+NH_2_)*. According to their model, the increase of *n(N)* and decrease of *n(O+NH_2_)* can enhance the corrosion inhibition effect of molecules. Among the positive effects is the presence of electron-donor group molecules, which increase electron density, and the polarity that can be responsible for the formation of a chelate on the metal surface. The negative effects are associated with the presence of electron-acceptor groups since these reduce the electron density.

Two indole derivatives were evaluated as corrosion inhibitors of C38 steel exposed to sulfuric acid. Two models were generated with a high correlation coefficient (R^2^) for the inverse of the charge transfer resistance (*R_ct_*). It was observed that the inhibition efficiency is mainly associated with *E_HOMO_* and *E_LUMO_* coefficients. The dipole moment was not relevant for the structure-activity relationship. Considering that *E_HOMO_* and *E_LUMO_* are important for chemical bond formation through electron sharing, the possibility of chemisorption processes taking place was deemed plausible, and hence, the contact between the metal and indole compounds was assumed to occur between the steel and pyridinium ions [23].

Gutierrez et al. [34] went over the analysis of 15 imidazole and benzimidazole derivatives as corrosion inhibitors of carbon steel. These researchers developed a mathematical model from which they were able to conclude that the inhibition efficiency is related to molecule volume, charge in nitrogen (q_N1_), electronegativity and aromaticity (bq^ISO^). The theoretical analysis was performed by DFT using the Perdew, Burke, and Ernzerhof (PBE) exchange-correlation functional, and the 6-311++G** orbital basis considered a useful functional-basis set combination in a wide variety of chemical systems. Table 6 shows the linear regression model, in which the dependent variable corresponds to the corrosion inhibition efficiency obtained by EIS at the maximum evaluated concentration of corrosion inhibitor. This mathematical model takes an aromaticity index (bq^ISO^) into account. This index is calculated with the Nucleus-Independent Chemical Shifts, where the data are obtained by means of a single-point calculation in a system formed by a test charge at a position of 1 Å away from the critical point of the aromatic ring. The obtained model was validated according to the methodology of Todeschini and co-workers [74].

Camacho-Mendoza et al. [35] gathered a collection of data from thirty organic compounds evaluated as corrosion inhibitors published in different articles (imidazole, benzimidazole, and pyridine derivatives). From the mathematical model generated, the researchers concluded that the index of aromaticity (anisotropy, bq^ANS^), electron donor capability (*ω*^−^) and molecular volume are all associated with the corrosion inhibitor efficiency.

A genetic function approximation (GFA) method along with the Becke–Lee–Yang–Parr (BLYP) exchange-correlation functional and the double numerical with polarization (DNP) basis set was used in a theoretical study of fourteen pyrimidine derivatives used to inhibit the corrosion of Aramco iron in 2.0 M HCl (data collected from literature). Among the advantages mentioned by the authors about the GFA are the construction of multiple models and the incorporation of Friedman’s lack-of-fit (LOF) error measure, which estimates the most appropriate number of features and resists the overfit. The final mathematical expression included the dipole moment (µ_D_) and energy gap (ΔE). The model achieved an R^2^ of 0.976 with an excellent fit of experimental and predicted data. It was found that the studied compounds inhibit iron corrosion by forming a molecular layer that decreases the iron dissolution [36].

The study of two series of thiosemicarbazides demonstrated that neither a simple correlation nor a direct trend relationship could be derived from the data in correlation with the inhibition performance, and their results were confirmed by two methods (PM3 and MNDO). According to the authors, the corrosion inhibition efficiency is a more complex process related to more than one quantum-chemical parameter. The correlation coefficient between experimental and calculated inhibition efficiencies may be improved significantly if a systematic change in the structure of the compounds is made [43].

Majid et al. [45] were responsible for the theoretical-experimental analysis of 2-mercaptobenzothiazole (mixed-type inhibitor), an N and S containing heterocyclic compound and interesting molecule in corrosion as it can form hydrophobic complexes with many metals such as iron, copper, cobalt, nickel, etc. The most important contribution in the report is the analysis of the change in the theoretical descriptors such as *E_HOMO_*, *E_LUMO_*, ΔE, μ_D_, hardness, softness, and Δ*N* of the different structures of 2-mercaptobenzothiazole (MBT). Considering that the MBT molecule exists in two tautomeric forms: thiole form (2-mercaptobenzothiazole; MBT; thio-enol) and thione form (benzothiazole-2-thione; BTT; thio-keto), an important effect on the chemical properties (electron-donating ability) was identified through the analysis of structural and electronic properties of cis-MBT, trans-MBT, BTT, cis-MBTH^+^, trans-MBTH^+^, and BTTH^+^ structures. The theoretical analysis of multi-heteroatoms-containing corrosion inhibitors (N and O) by Bahrami et al. [48] demonstrated a high correlation between the corrosion inhibition efficiency and *E_HOMO_*, *E_LUMO_*, ΔE, and μ_D_. An analysis of Mulliken population was used to identify the adsorption centres. The highest negative charges were found to be located on the nitrogen and oxygen atoms; however, no relation was found between the Mulliken charges and the inhibition efficiency. This result suggested that adsorption cannot be attributed to the formation of a chemical bond between the electronegative atoms and the metallic surface.

Among the group of multi-heteroatom inhibitors are the hydantoin derivatives (N and O heteroatom compounds) analysed by Olasunkanmi et al. [13]. A simple trend could not be found for most of the parameters with respect to inhibition efficiency. From the several linear and non-linear models studied, researchers found that the introduction of binding energy (BE) to each composite parameter leads to a better model. The best model obtained included the molecular weight (Mwt), frontier molecular orbitals energy gap, fraction of electron transferred and binding energy.

Five macrocyclic polyether compounds (n-MCTH) were studied by Lebrini et al. [18], these compounds include the N, S and O atoms mentioned previously by different authors as possible adsorption centres. The major structural differences among these molecules were due to the dihedral angle differences between the aromatic cycles and the thiadiazole frame (C–C–C–S). Changes in the structure were also responsible for changes in *E_HOMO_* and *E_LUMO_* energies. The linear model proposed by Bentis et al. [75] was applied to correlate the quantum-chemical parameters (*E_HOM_*_O_, *E_LUMO_*, and μ_D_) and the inhibitor concentration (C) with the experimental inhibition efficiencies (specifically, the charge transfer resistance, *R_t_*). The best linear regression equation for n-MCTH is enlisted in Table 6. The negative coefficient of *E_LUMO_* proves that the d orbitals of steel accept electrons from the macrocyclic compounds and feedback bonds form between steel and inhibitor molecules. The authors concluded that the corrosion inhibition efficiency can be explained not only in terms of electron orbital energies but also as a property dependent on the geometry of the molecules. The differences in inhibition performance might be determined by the number of oxygen atoms in the polyether ring, which is responsible for the increase in the molecular area and the adsorption ability of the inhibitor. The study of five sulphonamide derivatives as corrosion inhibitors of mild steel showed that both the *E_HOMO_* density and the f- function point to the fact that the sites with the highest tendency to donate electrons to metallic ions are the aromatic ring and the lone pair of electrons on the several heteroatoms of the sulphonamide compounds. Five quantum-chemical parameters were enough for the QSAR model to provide a strong correlation between theoretical and experimental inhibition efficiency. Two of the five non-linear models generated have been included in Table 6 [4].

To study the protection process of stainless steel produced from an iron-chromium-nickel alloy, Shojaie et al. [50] generated a QSAR model and applied it to the theoretical study of two pyrimidine compounds. A quadratic model was suggested to calculate the approximate inhibition efficiencies. According to the model generated, the regression analysis was a good fit for the experimental and calculated inhibition efficiency values of these pyrimidine derivatives. The following equation was proposed to predict the inhibition efficiency from the concentrations of the inhibitors and their quantum chemical parameters:

(28) $$IE_{Theor}\left(\%\right)=A+\frac{B_{1}X_{1}}{C_{1}}+\frac{B_{2}X_{2}}{C_{2}^{2}}+\frac{B_{3}X_{3}}{C_{3}^{3}}+\ldots \in +\frac{B_{n}X_{n}}{C_{n}^{n}}$$

In such equation, *A* and *B_n_* are constants obtained by regression, *X_n_* parameters are the quantum-chemical independent variables and *C_n_* corresponds to the inhibitor concentrations. With the aim to simply the mathematical models, these were constructed using only the first four terms. Table 6 includes one of the models generated for 5-Benzoyl-4-tolyl-6-phenyl-1,2,3,4-tetrahydro-2-hioxopyrimidine (BTPTT). The results showed that computational data for the liquid phase make a better representation of the experimental results than the theoretical results for the gas phase.

The study of corrosion inhibition of some amino acids (cysteine, glycine, leucine, and alanine) demonstrated that the inhibition mechanism involves the blockage of the metallic surface through physical adsorption. According to the Fukui indices, the Huckel charges, *E_HOMO_* and *E_LUMO_* establish that the adsorption sites are located at the carbonyl oxygen, the inhibition mechanism involves the donation electrons to Fe, as well as the formation of a feedback bond by the accepted electron from the lone pair of Fe. The formation of such a bond was looked at through a quantitative relationship between the *E_HOMO_*, *E_LUMO_* and the experimental inhibition efficiency. Table 6 displays three mathematical models from the analysis performed by Eddy et al. [51]. In the first equation obtained by PM6, the *E_HOMO_* coefficient is positive whereas that of *E_LUMO_* remains negative indicating that the formation of a feedback bond is favoured by increasing *E_HOMO_* and decreasing *E_LUMO_*. Non-linear models were generated in gas and aqueous phases including the examples provided in Table 6; a strong correlation between *IE_exp_* and *IE_Theor_* was found in all cases.

It is possible to predict a good or bad corrosion inhibitor by associating only one molecular descriptor. However, the activity of a molecule for the protection of a metallic surface is associated with different characteristics of its molecular structure. Furthermore, the corrosion inhibition efficiency is also determined by the conditions at which the experimentation is carried out. The generation of mathematical models with an appropriate number of descriptors, represents a good alternative to visualize some species as good corrosion inhibitors. Despite this, it is necessary to properly establish the experimental variables involved in the study, because the differences in the experimental conditions have an important impact on the corrosion inhibition efficiency values affecting the evaluation of better or worse inhibitors.

## 9. Perspectives

The corrosion process and the means to avoid it have constituted a research subject for various scientific groups over many years. Probably the main reason that encourages the study of the prevention of metal deterioration is associated with its economic impact.

Among the main methods to prevent corrosion in aqueous environments is the use of organic compounds. The main objective of several of the research groups interested in the study of corrosion inhibitors is to find those molecular properties that would increase their performance to delay or prevent this chemical phenomenon.

In addition to the identification of the highest performers to prevent the corrosion process, relevant factors such as economy, time and feasibility of analysis and, most relevantly, environmental protection, must be taken into account.

Technological advancement, the creation of more sophisticated computer software for analysis, together with experimental and mathematical techniques have allowed the creation of various mathematical models in which different molecular features have been considered to predict the corrosion inhibition percentages of a series of structurally similar molecules.

In spite of the advantages of the use of all the aforementioned strategies, it should be noted that there is a large number of mathematical models made from previously published databases. Hence, the comparison of the inhibitor performance can become a complicated, almost impossible, task. For the generation of the different inhibition models, each group of researchers has made a particular selection of the analysis conditions, among which we can mention: metal surface, type and concentration of the corrosive medium, temperature, exposure time, inhibitor concentration, techniques for experimental analysis (weight loss, electrochemical impedance spectroscopy and potentiodynamic polarization), and even some conditions of computational theoretical analysis such as the phase in which the molecular features are studied (gas or aqueous).

Due to the growing need to reduce the time and cost of research, the ideal conditions for the analysis of new corrosion inhibitor molecules should be established in a homogeneous manner. This would allow a wider panorama and reliable databases to obtain better, comparable results.

The homogeneous validation of the proposed mathematical models should also be taken seriously. As some authors have mentioned, it is common to fall into over-adjustment errors due to the excessive use of descriptors.

## Figures and Tables

**Table 1:** N-containing corrosion inhibitors (* illustrated inhibitor).

Example	Corrosion Inhibitor	Ef_WL_/Ef_PP_/Ef_EIS_	Ref.
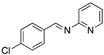	* (4-Chloro-benzylidene-pyridine-2-yl-amine)	99.5/99.6/99.6	[3]
	(4-Methyl-benzylidene)-pyridine-2-yl-amine	99.3/99.4/99.4	
	Benzylidene-pyridine-2-yl-amine	99.0/99.1/99.2	
Exp. C. N_CI_: 3, Met: mild steel, Media: HCl 1.0 M, Co: 2 × 10^−4^–1 × 10^−2^ M, t: 24 h (WL), T: 25 °C, TA: A			
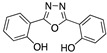	* 2,5-bis(2-hydroxyphenyl)-1,3,4-oxadiazole	98.2/94.7/99.1	[28]
	2,5-bis(2-pyridyl)-1,3,4-oxadiazole	95.3/91.5/98.6	
Exp. C. N_CI_: 2, Met: mild steel, Media: HCl 1.0 M, 0.5 M H_2_SO_4_, Co: 20–80 mg⋅L^−1^, t: 24 h (WL), 20 h (EIS), T: 30 °C, TA: NA			
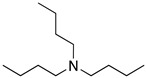	* Tributylamine	97.8/-/-	[29]
	Aniline	97.8/-/-	
	Sec-butylamine	67.5/-/-	
	Isopropylamine	63.6/-/-	
Exp. C. N_CI_: 23, Met: 13% Cr steel, Media: HCl (15%*w*/*v*), Co: 2%*w*/*v* inhibitor and 0.6% *w*/*v* formaldehyde (used to minimize hydrogen penetration), t: 3 h, T.: 60 °C, TA: A			
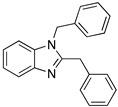	* 1,2-bisbenzylbenzimidazole	96	[30]
	2-Phenylbenzymidazole	95	
	Benzimidazole	29	
	Imidazole	17.5	
Exp. C. N_CI_: 11, Met: iron, Media: HCl 1.0 M. The analysis technique and the rest of the experimental conditions are not specified. TA: A			
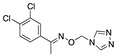	* 3,4-Dichloro-acetophenone-O-1′-(1′.3′.4′-triazolyl)-metheneoxime (DATM)	92.8/98.7/98.7	[25]
	4-Chloro-acetophenone-O-10-(10.30.40-triazolyl)-metheneoxime (CATM)	90.6/97.5/96.8	
	4-Fluoro-acetophenone-O-10-(10.30.40-triazolyl)-metheneoxime (FATM)	84.5/94.0/93.5	
Exp. C. N_CI_: 3, Met: mild steel, Media: HCl 1.0 M, Co:1 × 10^−5^–1 × 10^−3^ M, t: 3 h (WL), 30 min (PP), T: 25 °C, TA: A			
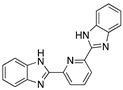	* 2,6-bis-(2-benzimidazolyl) pyridine (BBP)	-/94–96/97.0	[12]
	bis-(2-benzimidazolylmethyl) sulphide (BBMS)	-/91–92/96.0	
	bis-(2-benzimidazolylmethyl) oxide (BBMO)	-/89–91/94–95	
	1,2-bis-(2-benzimidazolyl) ethylene (BBE)	-/86–89/94.0	
Exp. C. N_CI_: 4, Met: mild steel, Media: HCl 1.0 M, Co: 0.1–1.0 mM, t: 1–96 h (WL), 45 min (PP), T.: 30 °C, TA: NA			
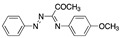	1-(4-Methyloxyphenylimino)-1-(phenylhydrazono)-propan-2-one	-/95.9/96.2	[31]
	1-(4-Methylphenylimino)-1-(phenylhydrazono)-propan-2-one	-/92.8/94.6	
	1-(4-Bromophenylimino)-1(phenylhydrazono) -propan-2-one	-/90.4/93.2	
	1-(4-Chlorophenylimino)-1(phenylhydrazono)-propan-2-one	-/88.3/91.3	
Exp. C. N_CI_: 5, Met: mild steel, Media: HCl 1.0 M, Co: 5 × 10^−6^–7.5 × 10^−5^ M, t: 30 min, T: 25 °C, TA: NA			
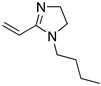	* 1-Butyl-2-propylene-2-imidazoline	95.1/-/-	[24]
	2-Phenylbenzimidazole	95.0/ - /-	
	1,2-Ethandiol	13.6/-/-	
	4-Hydroxybutylamine	4.8/-/-	
Exp. C. N_CI_: 34, Met: stainless steel, Media: HCl 5%, Co: ≈0.1 M, t: 48 h, T: T_R_ (room temperature), TA: A			
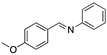	* Aniline, N-(p-methoxybenzylidene)	-/94.0/-	[32]
	Ethylenediamine, N,N-di(p-methoxybenzylidene)	-/90.0/-	
	Ethylenediamine, N,N’-dibenzylidene	-/89.0/-	
	Aniline, N-benzylidene	-/88.0/-	
Exp. C. N_CI_: 4, Met: aluminium, Media: HCl 2.0 N, Co: 10^−5^–10^−2^ M, t:-, T: 30 °C, TA: NA			
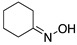	* Cyclohexanone oxime (CO)	93.9/98.4/95.2	[33]
	2-Butanone oxime (BO)	87.0/93.5/93.1	
	Acetone oxime (AO)	80.0//85.7/89.2	
Exp. C. N_CI_: 3, Met: aluminium, Media: HCl 1.0 M, Co: 0.2–2.0 mM, t: 2 h, T: 20–50 °C, TA: NA			
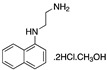	N-1-napththylethylenediamine dihydrochloride monomethanolate	90.2/88.1/87.9	[27]
Exp. C. N_CI_: 1, Met: carbon steel, Media: H_2_ SO_4_ 0.5 M, Co: 10^−5^–10^−2^ M, t: 2 h (WL), 30 min (PP), T: 25 °C, TA: NA			
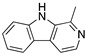	* 1-Methyl-9H-pyrido[3,4-b]indole	-/89.0/93.0	[23]
	9H-pyrido[3,4-b]indole	-/86.0/90.0	
Exp. C. N_CI_: 2, Met: C38 steel, Media: H_3_PO_4_ 2.0 M, Co:10^−5^–10^−2^ M, t: 2 h, 30 min, T: 30 °C, TA: A			
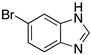	6-Bromo-1H-benzimidazole	-/-/89.4	[34]
	4-(Imidazol-1-yl)phenol	-/-/88.8	
	2-Chloro-1H-imidazole	-/-/57.1	
	2-Methylimidazole	-/-/30.2	
Exp. C. N_CI_: 15, Met: carbon steel, Media: HCl 1.0 M, Co:0.1–10 mM, t: 30 min, T: T_R_, TA: A			
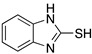	* 2-Mercaptobenzimidazole	-/88.7/90.4	[5]
	2-Methylbenzimidazole	-/57.1/76.3	
	Benzymidazole	-/52.2/73.8	
Exp. C. N_CI_: 3, Met: mild steel, Media: HCl 1.0 M, Co: 50–250 ppm, t: 30 min, T: 25 °C, TA: NA			
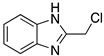	* 2-Chloromethylbenzimidazole	-/-/69.7	[35]
	2-Aminopyridine	-/-/51.2	
	2-Methylimidazole	-/-/13.9	
	Imidazole	-/-/12.5	
Exp. C. N_CI_: 6, Met: carbon steel, Media: HCl 1.0 M, Co: 1 × 10^−3^ M, t: -, T: T_R_, TA: A			
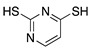	2-Mercaptopyrimidine	62.0/-/-	[7,36]
	* 2,4-Dimercaptopyrimidine	43.0/-/-	
	2,5,6-Triamino-3,4-dihydropyrimidine	−19.0/-/-	
	2-Mercapto-4-amino-5-nitro-1,6-dihydropyrimidine	−28.0/-/-	
Exp. C. N_CI_: 14, Met: Aramco iron, Media: HCl 2.0 M, Co: 1 × 10^−3^ M, t: 1 h, T: 40 °C, TA: A			

Simbology: TA: theoretical analysis, A: applied, NA: not applied, Exp. C.: experimental conditions; N_CI_: number of corrosion inhibitors, Co: inhibitor concentration, t: exposure time, T: temperature, Ef_WL_/Ef_PP_/Ef_EIS_: corrosion inhibition efficiencies obtained by weight loss (WL), potentiodynamic polarization (PP), and electrochemical impedance spectroscopy (EIS), respectively.

**Table 2:** S-containing corrosion inhibitors (* illustrated inhibitor).

Example	Corrosion Inhibitor	Ef_WL_/Ef_PP_/Ef_EIS_	Ref.
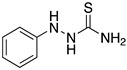	* 1-Phenyl thiosemicarbazide	-/100.0/-	[42,43]
	*p*-Amino acetophenone thiosemicarbazone	-/92.0/-	
	Acetophenone thiosemicarbazone	-/46.9/-	
	Thiosemicarbazide	-/43.8/-	
Exp. C. N_CI_: 10, Met: mild steel, Media: H_2_SO_4_, Co: 1 mM⋅dm^−3^, t: -, T: 30 °C. TA: A			
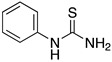	* Phenylthiourea (PTU)	-/-/97.2	[44]
	Methylthiourea (MTU)	-/-/95.6	
	Thiourea (TU)	-/-/92.9	
Exp. C. N_CI_: 3, Met: mild steel, Media: H_2_SO_4_ 0.1 M, Co: 1–10 mM, t:1 h, T: T_R_, TA: NA			
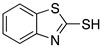	2-Mercaptobenzothiazole	-/97/-	[45]
Exp. C. N_CI_: 1, Met: Steel (API 5L X52), Media: H_2_SO_4_ 1.0 M, Co: 10^−4-^–10^−3^ M, t: 30 min, T: 25 °C, TA: A			
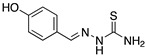	* 4-Hydroxybenzaldehyde thiosemicarbazone	-/97.0/90.0	[46]
	4-Hydroxy-3-methoxybenzaldehyde thiosemicarbazone	-/97.0/90.0	
	2-Indolecarboxaldehyde semicarbazone	-/81.0/86.0	
	2-Pyridinecarboxaldehyde semicarbazone	-/38.0/76.0	
Exp. C. N_CI_: 6, Met: carbon steel, Media: HCl 1.0 M, Co: 10^−4^–10^−2^ M, t: 30 min, T: 25 °C, TA: NA			
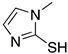	2-Mercapto-1-methylimidazole	90.4 /95.5/-	[47]
Exp. C. N_CI_: 1, Met: carbon steel, Media: HClO_4_ 1.0 M, Co: 7.5 × 10^−5^–2.5 × 10^−3^ M, t:1 h, T: 30 °C, TA: NA			

Simbology: TA: theoretical analysis, A: applied, NA: not applied, Exp. C.: experimental conditions; N_CI_: number of corrosion inhibitors, Co: inhibitor concentration, t: exposure time, T: temperature, Ef_WL_/Ef_PP_/Ef_EIS_: corrosion inhibition efficiencies obtained by weight loss (WL), potentiodynamic polarization (PP), and electrochemical impedance spectroscopy (EIS), respectively.

**Table 3:** O-containing corrosion inhibitors (*illustrated inhibitor).

Example	Corrosion Inhibitor	Ef_WL_/Ef_PP_/Ef_EIS_	Ref
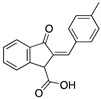	* 2-(4-Methylbenzylidene)-3-oxo-2,3-dihydro-1H-indene-1-carboxylic acid (MIC)	92.0/93.9/92.6	[21]
	2-Benzylidene-3-oxo-2,3-dihydro-1H-indene-1-carboxylic acid (BIC)	90.0/93.6/91.7	
	2-(Hydroxymethylene)-3,3-dimethyl-3-oxo-2,3-dihydro-1H-indene-1-one (HIO)	87.0/89.7/90.0	
Exp. C. NCI: 3, Met: mild steel, Media: HCl 1.0 M, Co: 10^−6^–10^−3^ M, t: 6 h, T: 30 °C, TA: NA			
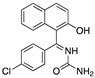	1-((4-Chlorophenyl)(2-hydroxynaphtalen-1-yl)(phenyl) methyl)urea (CPHU)	90/92.0/-	[48]
	1-((2-Hidroxy naphtalen-1-yl)(phenyl) methyl) urea (HNPU)	80/86.0/-	
	1-((2-Hidroxynaphtalen-1-yl)(4-methoxyphenyl)methyl)urea(HNMU))	75/67.0/-	
Exp. C. NCI: 3, Met: mild steel, Media: H_2_SO_4_ 0.5 M, Co: 2–10 ppm, t: 24 h (WL) and 30 min (PP), T: 20 °C, TA:A			
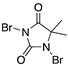	* 1,3-Dibromo-5,5-dimethylhydantoin (DBDMHYD)	-/89.8/91.4	[13]
	5-Methyl-5-phenylhydantoin (MPHYD)	-/88.1/84.6	
	1-Methylhydantoin (MHYD)	-/76.2/69.7	
	Hydantoin (HYD)	-/74.5/70.9	
Exp. C. NCI: 6, Met: mild steel, Media: HCl 0.5 M, Co: 10–50 ppm, t: 30 min, T: 30 °C, TA:A			
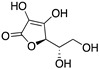	L-ascorbic acid	69.0/-/-	[49]
Exp. C. NCI: 1, Met: mild steel, Media: H_2_SO_4_ 0.01 M, Co: 10^−7^–10^−3^ M, t: -, T: T_R_, TA: NA			

Simbology: TA: theoretical analysis, A: applied, NA: Not applied, Exp. C.: experimental conditions; NCI number of corrosion inhibitors. Co: inhibitor concentration, t: exposure time, T: temperature, Ef_WL_/Ef_PP_/Ef_EIS_: corrosion inhibition efficiencies obtained by weight loss (WL), potentiodynamic polarization (PP), and electrochemical impedance spectroscopy (EIS), respectively.

**Table 4:** Multi-heteroatom containing corrosion inhibitors (* illustrated inhibitor).

Example	Corrosion Inhibitor	Ef_WL_/Ef_PP_/Ef_EIS_	Ref.
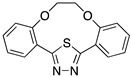	2,3,20,21-Dibenzo-4,7,10,13,16,19-hexaoxa-25-thia-23,24 diazabicyclo[20.2.1] pentacoza-21,24-diene (5-MCTH)	99.5/-/-	[18]
	2,3,17,18-Dibenzo-4,7,10,13,16-pentaoxa-22-thia-20,21-diazabicyclo [17.2.1]docosa-19,21-diene (4-MCTH)	99.2/-/-	
	2,3,11,12-Dibenzo-4,7,10-trioxa-16-thia-14,15 diazabicyclo[11.2.1]hexadeca-13,15-diene (2-MCTH)	99.0/-/-	
	* (2,3,8,9-Dibenzo-4,7-dioxa-13-thia-11,12-diazabicyclo[8.2.1]trideca-10,12-diene (1-MCTH)	97.7/-/-	
Exp. C. NCI: 5, Met: mild steel, Media: 1.0 M HCl, Co: 1 × 10^−6^–1 × 10^−4^ M, t: 24 h (WL), T: 30 °C, TA: A			
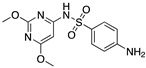	* Sulphadimethoxine	93.8/84.0/92.1	[4]
	Sulphanilamide (SNA)	89.6/87.6/82.1	
	Sulphisoxazole (SSZ)	88.4/88.4/88.5	
	Sulphamethoxazole (SMX)	88.6/89.8/86.4	
Exp. C. NCI: 5, Met: mild steel, Media:1.0 M HCl, Co: 1–5 × 10^−5^ M, t: 30 min, T: 30–50 °C, TA: A			
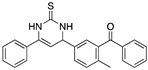	* 5-Benzoyl-4-tolyl-6-phenyl-1,2,3,4-tetrahydro-2-thioxopyrimidine	-/93.0/93.0	[14,50]
	5-Benzoyl-4-(4-carboxphenyl)-6-phenyl-1,2,3,4-tetrahydro-2-iminopyrimidine	-/90.0/90.0	
Exp. C. NCI: 2, Met: stainless steel, Media:1.0 M HCl, Co:1 × 10^−4^–5 × 10^−3^ mol⋅dm^−3^, t: 30 min, T: 25 °C. The experimental data were obtained by Caliskan, et al. [14]. TA: A			
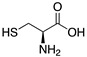	* L-Cysteine	82.2/-/-	[51]
	L-Leucine	64.4/-/-	
	L-Alanine	56.6/-/-	
	Glycine	56.5/-/-	
Exp. C. NCI: 4, Met: mild steel, Media:0.1 M HCl, Co: 0.1–0.5 g⋅L^−1^, t: 24, 168 h, T: 30, 60 °C, TA: A			
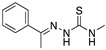	* (E)-N-methyl-2-(1-phenylethylidene)hydrazinecarbothioamide PHCARB	80.7/-/-	[22]
	2-(Diphenylmethylene)-N-phenylhydrazinecarbothioamide DPHCARB	74.6/-/-	
	(E)-2-(2-hydroxy-1,2-diphenylethylidene)-N-phenylhydrazinecarbothioamide HDPPCARB	44.0/-/-	
	(E)-2-(2-oxo-1,2-diphenylethylidene)-N-phenylhydrazinecarbothioamide ODPPCARB	36.0/-/-	
Exp. C. NCI: 4, Met: mild steel, Media:0.1 M HCl, Co: 1–5 × 10^−4^ M, t: 168 h, T: 30 °C, TA: A			
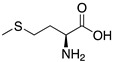	Methionine	72.7/84.6/85.8	[10]
	Arginine	66.9/52.9/57.4	
	Glycine	1.6/1.4/13.9	
	Serine	−1.2/−50/−61.6	
Exp. C. NCI: 16, Met: Armco iron, Media: HCl 1.0 M, Co: 10^−3^ M, et: 6 h (WL), 10 y 30 min (PP), T: 35 °C, TA: A			

Simbology: TA: theoretical analysis, A: applied, NA: not applied, Exp. C.: experimental conditions; NCI: number of corrosion inhibitors. Co: inhibitor concentration, t: exposure time, T: temperature, Ef_WL_/Ef_PP_/Ef_EIS_: corrosion inhibition efficiencies obtained by weight loss (WL), potentiodynamic polarization (PP), and electrochemical impedance spectroscopy (EIS), respectively.

**Table 5:** Examples of descriptors used in the theoretical analysis of corrosion inhibitors.

Quantum Descriptor	Symbol	Description	Reference
Energy of the highest occupied molecular orbital	E_HOMO_	Associated with the electron-donating ability of a molecule.	[3]
Energy of the lowest unoccupied molecular orbital	E_LUMO_	Indicates the ability of the molecule to accept electrons.	[47]
Ionization potential	IP	It is a descriptor of the chemical reactivity of atoms and molecules. *IP* is the minimum energy required to remove an electron from an atom.	[50]
Electron affinity	A	It is a property that determines how susceptible a molecule is towards the attack of a nucleophile.	[53]
Dipole moment	μ_D_	The dipole moment is considered a measure of the stability of the formed complex on a metal surface. It is an indicator of the asymmetry in the molecular charge distribution. It is related to the hydrophobic character of the molecules.	[43,48,54,55]
Energy gap	ΔE	ΔE= *E_HOMO −_ E_LUMO_* It has been mentioned that the successful adsorption and proper efficiency of an inhibitor have been characterized by a high *E_HOMO_,* a low *E_LUMO_*_,_ and a small energy gap. The lower the ΔE is, the higher the stability of the metal-inhibitor interaction.	[12,54,56]
Fraction of electron transferred	Δ*N*	If Δ*N* > 3.6, the inhibition efficiency increases.	[12]
Global hardness	*η*	It is a parameter associated with the resistance of an atom to transfer its charge.	[12]
Softness	σ	The σ shows the reactivity of the inhibitor molecules in terms of charge transfer.	[12]
Electronegativity	χ	The electronegativity measures the power of a group of atoms to attract electrons towards itself.	[12]
Electrophilicity index	ω	It is a reactivity descriptor that allows the quantitative classification of the global electrophilic nature of a molecule. This descriptor has been proposed as a measure of energy lowering due to maximal electron flow between donor and acceptor.	[25,57]
Electrodonating power	ω^-^	A descriptor associated with the ability of a species to donate electrons.	[58]
Electroaccepting power	ω^+^	A descriptor associated with the ability of a species to accept electrons.	[58]
Dipole polarizability	α	α is a measure of the mean polarizability. Higher values of α enable a strong adsorption process.	[25]
Fukui functions	*f_k_^+^*, *f_k_^−^*	These functions indicate the part of the molecule where nucleophilic, electrophilic, and radical attack is most likely to occur.	[53]
Partition coefficient	Log P	Log P is a hydrophobic parameter of a molecule.	[59]

**Table 6:** Examples of mathematical models generated for corrosion analysis.

Molecules Evaluated	Theoretical Calculation	Mathematical Model	*R^2^*	Ref.
Schiff base	AM1 semi-empirical method	$IE_{exp}\left(\%\right)=2.084E_{HOMO}-3.041E_{LUMO}+115.772$	1.00	[3]
		$IE_{Theor}=\frac{\left(-604.90E_{HOMO}+5864.86E_{LUMO}+1190.06\mu_{D}-642.67\right)C}{\left[1+\left(-604.90E_{HOMO}+5864.86E_{LUMO}+1190.06\mu_{D}-642.67\right)C\right]}$	0.98	
Amines, thioureas, acetylenic alcohols	AM1 methodology was used for most descriptors, PC model provided the volume calculations	$\begin{array}{c} lnK_{ads}=-0.93N-7.64P+6.74C-2.27C_{12}+0.94C_{13}-1.06C_{1}\\ -7.22C_{2}-2.17E_{HOMO}-1.17DP+7.45V-1.15A_{1}\\ -1.81A_{2}+7.12NCS-1.869NOH-1.085NCR \end{array}$	0.98	[29]
		$lnK_{ads}=1.52M-0.79P+0.53C_{12}+0.80NT-0.66NOH$	0.98	
		$\begin{array}{c} lnK_{ads}=-2.688\times 10^{-2}A_{1}+0.115A_{2}+4.530\times 10^{-2}A_{3}+8.762\times 10^{-2}NB-1.305\times 10^{-2}NC\\ +0.102NCS-4.518\times 10^{-2}NT-5.172\times 110^{-2}NOH-1.099\times 10^{-3}NCR+4.996\times 10^{-2}NR\\ -6.338\times 10^{-2}N-0.114ED+0.184M+0.180P-6.838\times 10^{-2}C+8.092\times 10^{-2}C_{12}\\ -4.783\times 10^{-2}C_{13}-1.130\times 10^{-2}C_{14}-0.167C_{1}-0.152C_{2}-0.122E_{HOMO}\\ -7.838\times 10^{-2}E_{LUMO}-7.004\times 10^{-3}\Delta E+0.109\mu +0.141V \end{array}$	0.85	
Triazole derivatives	Density Functional Theory (DFT), B3LYP/6-31G	$IE_{Theor}=\frac{\left(18.38E_{HOMO}-7.28E_{LUMO}-0.012V+123.36\right)C}{\left[1+\left(18.38E_{HOMO}-7.28E_{LUMO}-0.012V+123.36\right)C\right]}$ Derived QSAR equation in gas phase	0.94	[25]
		$IE_{Theor}=\frac{\left(-2.19E_{HOMO}-1.24E_{LUMO}-0.014V-10.94\right)C}{\left[1+\left(-2.19E_{HOMO}-1.24E_{LUMO}-0.014V-10.94\right)C\right]}$ Derived QSAR equation in aqueous phase	0.95	
Imidazole derivatives	Restricted Hartree–Fock level (RHF) using MINDO/3, MNDO, PM3 and AM1 semi-empirical SCF-MO methods.	$IE_{exp}\left(\%\right)=1174.95+214.612E_{HOMO}-16.793E_{LUMO}$ Gas phase (series 1)	0.90	[30]
		$IE_{exp}\left(\%\right)=517.7+53.8E_{HOMO}-1.97E_{LUMO}$ Aqueous phase (series 1)	0.82	
		$IE_{exp}\left(\%\right)=2420.86+295.67E_{HOMO}-30.08E_{LUMO}$ Gas phase (series 2)	0.97	
		$IE_{exp}\left(\%\right)=601.53+63.07E_{HOMO}-1.405E_{LUMO}$ Aqueous phase (series 2)	0.99	
Imidazole and benzimidazole derivatives	---	$IE_{exp}\left(\%\right)=38.47+20.21n\left(N\right)-7.98N\left(O+NH_{2}\right)+14.94\eta^{+}-17.93\eta^{-}$	0.97	[24]
Indole derivatives	DFT, B3LYP functional and 6-31G(2d,2p) basis	$R_{ct}=150+\left(-359539E_{HOMO}+1585825E_{LUMO}\right)C$	0.97	[18]
		$R_{ct}=150+\left(-402535E_{HOMO}+960146E_{LUMO}\right)C$	0.97	
Imidazole and benzimidazole derivatives	DFT: PBE/6-311++G **	$IE_{exp}\left(\%\right)=5130.95-32.03\chi +533.4bq^{ISO}+0.37V+1433.78q_{N1}$	0.92	[35]
Imidazole, benzimidazole and pyridine derivatives	DFT: PBE/B3LYP/M06, using the orbital basis 6-31G* and 6-311++G**	$IE_{exp}\left(\%\right)=92.965+0.152V+35.337\omega^{-}+3.592bq_{ANS}$	0.75	[34]
Pyrimidine derivatives	DFT BLYP/DNP.	$IE_{exp}\left(\%\right)=-4.324\mu -46.527\Delta E+376.4$	0.98	[36]
Thiosemicarbazides	PM3 and MNDO method.	$IE_{Theor}=\frac{\left(-6.7E_{HOMO}-5.9E_{LUMO}-3.5\mu -43.7\right)C}{\left[1+\left(-6.7E_{HOMO}-5.9E_{LUMO}-3.5\mu -43.7\right)C\right]}\times 100$	0.84	[43]
		$IE_{Theor}=\frac{\left(227.2+23.5E_{HOMO}-3.8\mu \right)C}{\left[1+\left(227.2+23.5E_{HOMO}-3.8\mu \right)C\right]}\times 100$	0.92	
Thiosemicarbazides/ Thiosemicarbazones	Data obtained from literature	$IE_{exp}=\frac{e^{-2.5219E_{HOMO}-0.5119\mu_{D}-18.1761}C}{\left(1+e^{-2.5219E_{HOMO}-0.5119\mu_{D}-18.1761}C\right)}$	0.89	[42]
		$IE_{exp}=\frac{e^{263.12\Delta E-17.26\Delta E^{2}-999.22}C}{\left(1+e^{263.12\Delta E-17.26\Delta E^{2}-999.22}C\right)}$	0.88	
2-Mercaptobenzothiazole	DFT: B3LYP/6-31+G*	$IE_{Theor}=\frac{\left(2.23E_{HOMO}-8.37E_{LUMO}+5.47\Delta E+1.76\Delta N+6.47\mu +119.87V+2.07\right)C}{\left[1+\left(2.23E_{HOMO}-8.37E_{LUMO}+5.47\Delta E+1.76\Delta N+6.47\mu +119.8V+2.07\right)C\right]}\times 100$	0.96	[45]
Urea derivatives	DFT: B3LYP/6-31G	$\Delta E=-0.0024IE_{exp}-4.3145$	0.95	[48]
		$E_{HOMO}=-0.0107IE_{exp}-4.8596$	0.928	
Hydantoin derivatives	DFT: B3LYP/6-31G+(d,p)	$IE_{Theor}=129.054-0.181Mwt-6.550\Delta E-174.884\Delta N+0.484BE$	1.00	[13]
		$IE_{Theor}=\frac{\left(9.1\times 10^{13}Mwt+4.9\times 10^{14}\Delta N+9.7\times 10^{13}\Delta E+2.4\times 10^{13}BE-5.9\times 10^{13}\right)}{\left(1+1.8\times 10^{13}Mwt+1.8\times 10^{16}\Delta N+8.1\times 10^{14}\Delta E-1.2\times 10^{13}BE-7.2\times 10^{15}\right)}$	0.99	
Polyether compounds	DFT B3LYP/6-31G(d,p)	$R_{t}=5+\left(2.710^{7}E_{HOMO}-7.610^{7}E_{LUMO}+3.910^{6}\mu \right)C$	0.81	[18]
Sulphonamide derivatives	DFT: B3LYP/6-311+G(d,p)	$IE_{exp}\left(\%\right)=\frac{\left(2.47\omega +8.56\times 10^{-2}E_{LUMO}-5.27\times 10^{-2}\mu +6.20\eta -4.11\times 10^{-2}LogP-21.81\right)*5000}{\left(1+\left(2.47\omega +8.56\times 10^{-2}E_{LUMO}-5.27\times 10^{-2}\mu +6.20\eta -4.11\times 10^{-2}LogP-21.81\right)*50\right)}$	1.00	[4]
		$IE_{exp}\left(\%\right)=\frac{(-1.44\Delta E+3.48E_{LUMO}-3.50E_{HOMO}+1-07\eta +1.75\omega^{+}-17.85)*5000}{\left(1+\left(-1.44\Delta E+3.48E_{LUMO}-3.50E_{HOMO}+1-07\eta +1.75\omega^{+}-17.85\right)*50\right)}$	0.99	
Pyrimidine compounds	B3LYP/6-311++G(d,p)	$IE_{Theor}\left(\%\right)=9.255\times 10^{1}-3.213\times 10^{-3}\frac{\chi }{C_{1}}+3.432\times 10^{-7}\frac{\mu }{C_{2}^{2}}-1.768\times 10^{-10}\frac{\Delta E}{C_{3}^{3}}-5.859\frac{E_{HOMO}}{C_{4}^{4}}$ * equation for BTPTT, phase gas	0.98	[14]
		$IE_{Theor}\left(\%\right)=9.326\times 10^{1}+6.249\times 10^{-4}\frac{E_{HOMO}}{C_{1}}-1.093\times 10^{-7}\frac{E_{LUMO}}{C_{2}^{2}}$	0.99	
Amino acids	PM6, PM3, MNDO and RM1 for semi-empirical studies. DFT. Local selectivity	$IE_{exp}=125.80E_{HOMO}-121.83E_{LUMO}+1402.96$ PM6 Hamiltonian	0.93	[51]
		$IE_{Theor}=\frac{\left(1.085E_{HOMO}+1.114E_{LUMO}+\Delta E+4.128\mu \right)C}{\left[1+\left(1.085E_{HOMO}+1.114E_{LUMO}+\Delta E+4.128\mu \right)C\right]}\times 100$ AM1 Hamiltonian, gas phase	0.86	
		$IE_{Theor}=\frac{\left(0.896E_{HOMO}+1.38E_{LUMO}+\Delta E+\mu +1.649E_{diel}+18.38\right)C}{\left[1+\left(0.896E_{HOMO}+1.38E_{LUMO}+\Delta E+\mu +1.649E_{diel}+18.38\right)C\right]}\times 100$ RM1 Hamiltonian, aqueous phase	0.96	
Carbozones	AM1, PM6, PM3, MNDO and RM1 Hamiltonians. Correlation MP2, basis STO-3G	$IE_{exp}\left(\%\right)=-14.686E_{HOMO}-48.966$	0.94	[22]
		$IE_{Theor}=\frac{\left(1.0176E_{HOMO}+0.9743E_{LUMO}+1.0351\Delta E+CosA+CosV+428.6731\right)C}{\left[1+\left(1.0176E_{HOMO}+0.9743E_{LUMO}+1.0351\Delta E+CosA+CosV+428.6731\right)C\right]}$	0.83	
Simbology Austin Model 1: AM1, Modified Intermediate Neglect of Differential Overlap: MINDO, Modified Neglect of Diatomic Overlap: MNDO, Parametric Method 3: PM3, Self consistent field molecular orbital: SCF-MO				
